# Palladium-Catalyzed C–H Arylation of Benzofurans with Triarylantimony Difluorides for the Synthesis of 2-Arylbenzofurans

**DOI:** 10.3390/molecules26010097

**Published:** 2020-12-28

**Authors:** Yuki Kitamura, Yuki Murata, Mizuki Iwai, Mio Matsumura, Shuji Yasuike

**Affiliations:** School of Pharmaceutical Sciences, Aichi Gakuin University, 1-100 Kusumoto-cho, Chikusa-ku, Nagoya 464-8650, Japan; ag183a01@dpc.agu.ac.jp (Y.K.); y-murata@dpc.agu.ac.jp (Y.M.); colors1120zuky@gmail.com (M.I.); m-matsu@dpc.agu.ac.jp (M.M.)

**Keywords:** C–H arylation, palladium catalyst, antimony, triarylantimony difluoride, benzofuran

## Abstract

Pd-catalyzed regioselective C–H arylation is a useful tool for the chemical modification of aromatic heterocycles and 2-arylbenzofuran derivatives are of interest as biologically active substances. Herein, the reaction of triarylantimony difluorides with benzofurans under aerobic conditions in 1,2-DCE, using 5 mol% Pd (OAc)_2_ and 2 eq. of CuCl_2_ at 80 °C, produced a variety of 2-arylbenzofurans in moderate-to-high yields. The reaction is sensitive to the electronic nature of the substituents on the benzene ring of the triarylantimony difluorides: an electron-donating group showed higher reactivity than an electron-withdrawing group. Single crystal X-ray analysis of tri(*p*-methylphenyl) antimony difluoride revealed that the central antimony atom exhibits trigonal bipyramidal geometry.

## 1. Introduction

**Figure 1:**
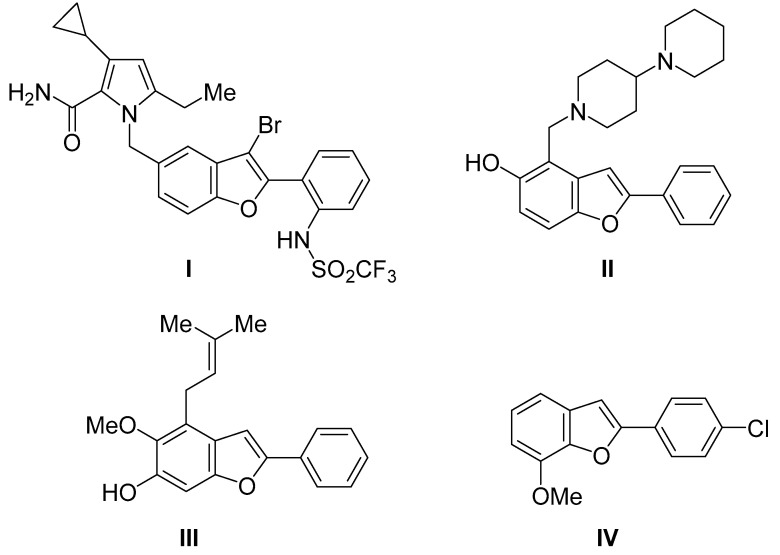
Biologically active 2-arylbenzofuran derivatives.

Benzo[*b*]furans are important heterocycles because they are key skeletons in many natural products, bioactive compounds, and organic materials [1,2,3,4,5,6]. 2-Arylbenzofuran derivatives in particular have attracted significant interest because of their potential as biological and pharmaceutical therapeutic agents [7]. For example, saprisartan (**I**) [8,9] acts as an angiotensin II receptor antagonist and renin-angiotensin system inhibitor, compound **II** displays potent hepatoprotective and antioxidant activities [10], compound **III** shows antitumor activity on a panel of human cancer cell lines [11], and compound **IV** has promotive activity on estrogen biosynthesis [12] (Figure 1).

**Scheme 1:**
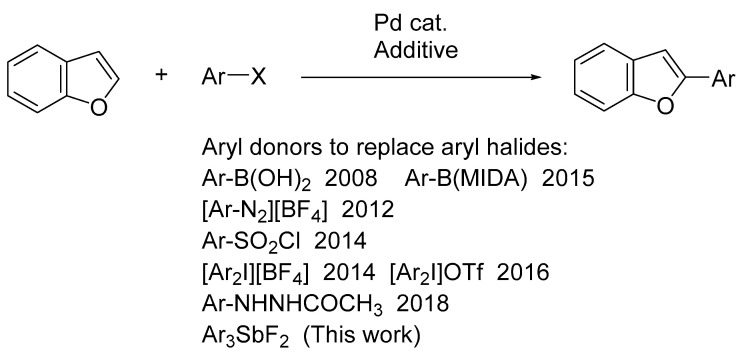
Pd-catalyzed coupling reaction of benzofuran with aryl donors.

Many methods have been developed for the synthesis of 2-arylbenzofurans. Pd-catalyzed direct C–H arylation of 5-membered heteroaromatic compounds is one of the most powerful tools for the synthesis of arylated heterocycles [13,14]. 2-Arylbenzofurans have been prepared in this way using various aryl group donors. In 1990, Ohta et al. reported that the C–H arylation of benzofuran using arylbromides in the presence of catalytic Pd(PPh_3_)_4_ and excess amount of potassium acetate gave 2-phenylbenzofuran in 23% yield [15]. Since then, the reaction conditions using aryl halides have been improved in order to obtain 2-arylbenzofurans more efficiently [16,17,18,19,20]. Reactions using other aryl donors to replaces the aryl halides have also been developed (Scheme 1). Shi et al. demonstrated the coupling of benzofuran with phenylboronic acid and Cu(OAc)_2_ using a Pd(OAc)_2_ catalyst in trifluoroacetic acid [21]. Wu and Huang found that the reaction of benzofuran and aryl *N*-methyliminodiacetic acid (MIDA) boronates proceeds in the presence Pd-alkoxy catalysts and benzoquinone under acidic conditions [22]. Correia et al. proved that Pd(OAc)_2_ catalyzes the arylation of benzofuran with aryldiazonium tetrafluoroborates [23]. Doucet et al. reported the reaction of benzofurans and aryl sulfonyl chlorides in the presence of Li_2_CO_3_ [24,25]. Glorius et al. revealed that 2-arylation of benzofuran with diphenyliodonium tetrafluoroborate progresses using a heterogeneous Pd–C catalyst [26]. In addition, McGlacken and Wan independently reported the use of PdO-Fe_3_O_4_ and a solid-based Pd catalyst for the reaction of benzofuran and diaryliodonium salts [27,28]. Tang et al. demonstrated the C–2 arylation of benzofurans using *N’*-acyl arylhydrazines in the presence of a PdCl_2_(MeCN)_2_ catalyst and the 2,2,6,6-tetramethylpiperidine 1-oxyl radical (TEMPO) [29]. These procedures could be used complementarily; however, each reaction has several drawbacks in substrate scope and efficiency. Therefore, the development of an effective novel aryl donor and the elucidation of its reactivity for the C–H arylation of benzofuran derivatives are required. Pentavalent organoantimony compounds such as triarylantimony diacetates [Ar_3_Sb(OAc)_2_] and tetraphenylantimony acetate [Ph_4_Sb(OAc)] could be used as pseudo-halides for Pd-catalyzed C–C(Ar) bond formation in Heck- [30,31,32], Stille- [33], Hiyama- [34], Suzuki- [35,36] and Sonogashira-type reactions [37]. We also recently reported that triarylantimony difluorides (Ar_3_SbF_2_) serve as an arylating reagent for the Pd-catalyzed β-selective C–H arylation of thiophenes [38]. Ar_3_SbF_2_ is an easy-to-handle compound that can be stored under air and is known to have low toxicity [39,40]. Inspired by the aforementioned reports and a continuation of our studies on the C–H arylation of aromatic heterocycles, this paper presents the Pd-catalyzed regioselective arylation of benzofurans with Ar_3_SbF_2_ for the synthesis of 2-arylbenzofurans.

## 2. Results and Discussion

### 2.1. Pd-Catalyzed C–H Arylation of Benzofurans with Triarylantimony Difluorides

We previously reported the Pd-catalyzed β-selective C–H arylation of benzothiophene with Ar_3_SbF_2_ in the presence of 5 mol% Pd(OAc)_2_ and 2 eq. CuCl_2_ in 1,2-DCE at 80 °C under aerobic conditions [38]. In order to suitably investigate the reactive arylating agent, various pentavalent organoantimony compounds **2a**–**12** (0.5 mmol) were reacted with benzofuran **1a** (0.5 mmol) under the above optimal experimental conditions. The results are summarized in Table 1. Ph_3_SbF_2_ **2a** and Ph_3_SbCl_2_ **3** gave 2-phenylbenzofuran **13a** in over 80% yields (entries 1, 2). Ph_3_SbF_2_ **2a** was found to be the best arylating agent for the reaction in terms of the yield and reaction time (entry 1). It is also noteworthy that **2a** has the advantage of not producing biphenyl as a by-product. Moreover, this reaction afforded only 2-phenylbenzofuran **13a**, and 3-phenylbenzofuran could not detect using gas chromatography (GC). The reaction of **1a** and **2a** was carried out in a 3:1 ratio because **2a** has three phenyl groups. However, the yield was low (28%), which showed that only one of the three phenyl groups on antimony reacts in the C–H arylation. Aryl donors such as previously known aryl halides, [15,16,17,18,19,20] arylboron reagents, [21,22] and aryl sulfonyl chlorides [24,25] often require bases, ligands for Pd catalyst, oxidants, high temperature, inert atomosphere, and/or acidic conditions. In contrast, Ph_3_SbF_2_ is similar to arylhydrazines [29] and much more stable than the aryldiazonium salt [23], and the reaction proceeds smoothly only by adding an oxidant under mild conditions. However, Ph_3_SbF_2_ has a low atom economy similar to the hypervalent iodine reagents, [26,27,28] and only one of the three aryls can be used in the reaction.

To demonstrate the efficiency and generality of this C–H arylation, the reactions of various benzofurans **1** (0.5 mmol) and Ar_3_SbF_2_ **2** (0.5 mmol) were investigated under aerobic conditions in 1,2-DCE using Pd(OAc)_2_ (5 mol%) as a catalyst at 80 °C. The results are summarized in Table 2. Antimony compounds **2b**–**j** used for the reactions were easily synthesized by the oxidative fluorination of triarylstibanes (Ar_3_Sb) with nitrosyl tetrafluoroborate using our method [40]. The reactions of benzofuran **1a** with Ar_3_SbF_2_ **2b**–**h** proceeded selectively at the α-position to give coupling products **13b**–**g** in 19–91% yields, with the exception of nitrile derivative **13h**. In the case of **2b** having methoxy group, the disappearance of starting materials was observed within a short time (1 h), but there was a low yield of the corresponding **13b** (19%). When the reaction was carried out at 40 °C, **13b** was obtained in 60% yield. The use of compounds **2g** and **2h**, which have trifluoromethyl and cyano groups, respectively, resulted in low yield or did not react. These results suggest that this reaction is affected by the electronic nature of the substituent on the phenyl ring of Ar_3_SbF_2_, and that electron-donating groups have superior reactivity. In addition, sterically hindered ortho-substituted Ar_3_SbF_2_ **2i** gave coupling product **13i** without difficulty. There are few reports of the Pd-catalyzed C–H arylation of benzofuran bearing an electron-donating or electron-withdrawing substituent at the 5-position [19,20,29]. The reactions of various benzofurans **1** with Ph_3_SbF_2_ **2a** afforded the corresponding 2-phenylbenzofurans **13j**–**n** in moderate-to-excellent yields. 5-Methoxybenzofuran **1b** bearing an electron-donating group showed a slightly lower reactivity in these reactions. However, unlike substituted on the phenyl ring of Ar_3_SbF_2_, the difference in the electronic nature of the substituents in 5-substituted benzofurans **1** was not virtually reflected in the reaction time and yields. Double C–H arylation of benzofuran **1a** was attempted using 2 eq. of Ph_3_SbF_2_, but the corresponding 2,3-diphenylbenzofuran **13o** was not obtained, and 2-phenylbenzofuran **13a** was isolated in 99% yield. **13o** was not obtained even in the reaction of 2-phenylbenzofuran **13a** with **2a**. Finally, benzoheteroles containing other chalcogen elements such as selenium and tellurium were reacted with **2a**, but the reaction did not proceed and the corresponding α- or β-arylated products **13p** and **13q** were not produced.

**Figure 2:**
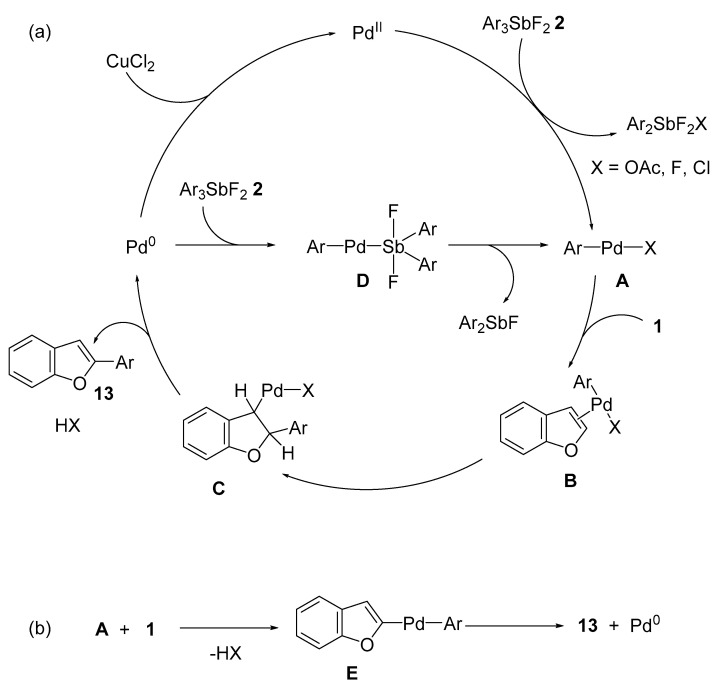
Possible mechanisms.

At present, the reaction mechanism is unclear. We consider that the mechanism would be similar to that of the C–H arylation of benzofuran with MIDA boronates and benzenesulfonyl chlorides proposed by Wu and Doucet et al., respectively, for the synthesis of 2-arylbenzofurans [22,24]. Additionally, Gushchin et al. reported the Heck-type *C*- arylation of methyl acrylate and alkenes with pentavalent organoantimony compounds such as Ar_3_Sb(OAc)_2_ and Ph_4_SbOAc [31,32]. Possible mechanisms for the C–H arylation of Ar_3_SbF_2_ with benzofurans are depicted in Figure 2. The initial step is the transmetalation of Pd(II) with Ar_3_SbF_2_ **2** to afford Ar-Pd-X **A** with liberation of Ar_2_SbF_2_X [31]. Coordination of benzofuran to complex **A** leads to the generation of complex **B**, which undergoes arylpalladation of the benzofuran to give intermediate **C** (Figure 2a). β-Hydride elimination and reductive elimination form coupling product **13** and HX, and generate a Pd(0) species. The Pd(0) species is oxidized by the copper regent, and Pd(II) is regenerated. An alternative pathway would involve the oxidative addition of Ar_3_SbF_2_ to the Pd(0) species to give ArPdSb complex **D**, which is then transformed to complex **A** and Ar_2_SbF [31]. By-products such as Ar_2_SbF and Ar_2_SbF_2_X that would be released in this reaction have not been confirmed or isolated at this point. An alternative pathway is also under consideration (Figure 2b); electrophilic aromatic substitution and/or concerted metalation-deprotonation proceeding from intermediate **B** and benzofuran to form complex **E** are also conceivable. Intermediate **E** undergoes reductive elimination to afford coupling product **13**.

### 2.2. X-ray Crystal Structure of Triarylantimony Difluoride

**Figure 3:**
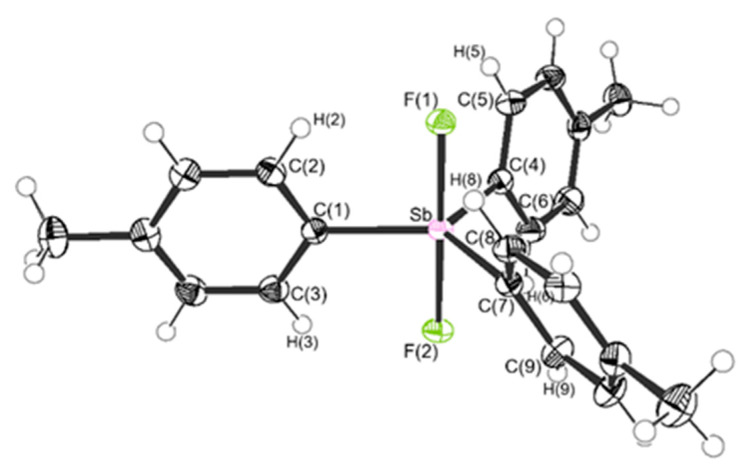
ORTEP drawing of *p*-Tol_3_SbF_2_ **2c** with 50% probability.

The molecular structure of *p*-Tol_3_SbF_2_ **2c** was determined by single crystal X-ray diffractometry. The structure is depicted in Figure 3, and selected geometrical parameters are shown in Table 3. The hydrogen atoms were placed according to the electron density in calculated positions and were included in the refinement. The central antimony atom of **2c** adopted a trigonal bipyramidal structure. The three carbon atoms on the phenyl ring [C(1), C(4), and C(7)] lie in the equatorial plane; fluorine atoms F(1) and F(2) occupy the axial positions in the trigonal bipyramidal structure. The sum of the angles in the equatorial plane [C(1)–Sb–C(4), C(1)–Sb–C(7), and C(4)–Sb–C(7)] is 360°, and axial angle [F(1)–Sb–F(2)] is 178.93°. Moreover, the angles of F(1)–Sb–C and F(2)–Sb–C are almost 90°: 89.19–90.83°. Notably, intramolecular hydrogen bonds of the type C–H∙∙∙F involving the six hydrogens at the *ortho* positions of the three tolyl groups are observed. The atomic distances of H∙∙∙F {2.28(3)–2.50(2)} are significantly smaller than the sum of the van-der-Waals radii of hydrogen and fluorine (2.55 Å) [41]. The result of these intermolecular interactions is that the three benzene rings have sterically strained conformations that are close to the eclipsed conformation with the axial Sb–F bonds; the dihedral angles of F-Sb(7)–C(ipso)–C(Ar) in compound **2c** are remarkably small and narrow range (6.71° to 25.99°).

## 3. Materials and Methods

### 3.1. General

Melting points were measured on a Yanagimoto micro melting point hot stage apparatus (Yanaco, Kyoto, Japan) and are uncorrected. ^1^H-NMR (400 MHz, CHCl_3_: δ: 7.26 ppm as an internal standard), ^13^C-NMR (100 MHz, CDCl_3_: δ: 77.00 ppm as an internal standard) and ^19^F-NMR (376 MHz, PhCF_3_: δ: −64.0 ppm as an external standard) spectra were recorded on ECZ-400S spectrometer (JEOL, Tokyo, Japan) in CDCl_3_. IR spectra were recorded on a FTIR-8400S spectrophotometer (Shimadzu, Kyoto, Japan) and reported in terms of frequency of absorption (cm^−1^). Only selected IR bands are reported. Mass spectra were obtained on a JEOL JMP-DX300 instrument (70 eV, 300 mA). Column chromatography was performed on Silica Gel 60N (Kanto Chemical Co., Inc. (Tokyo, Japan)). Reagents were purchased from Wako Pure Chemical Industries, Ltd. (Osaka, Japan), Tokyo Kasei Kogyo Co., LTD (Tokyo, Japan) and SIGMA-ALDRICH Japan K.K. (Tokyo, Japan) and used without further purification.

### 3.2. General Procedure for the C–H Arylation

Ar_3_SbF_2_ (**2**) (0.5 mmol), Pd(OAc)_2_ (0.025 mmol), CuCl_2_ (1.0 mmol) and benzofuran derivative (**1**) (0.5 mmol) were added to 1,2-dichloroethane (3.0 mL) in a round-bottom flask. After stirring at 80 °C for 3–72 h, the mixture was cooled to room temperature and filtered through a short plug of Celite. The Celite plug was flushed with CH_2_Cl_2_, and the filtrate was evaporated to dryness under reduced pressure. The crude product was purified on a silica gel column chromatography to give the desired product **13a**–**n** (**13a**–**c**, **i**, **k**: [19], **13d**, **g**, **j**: [20], **13e**, **l**: [29], **13f**: [42], **13m**: [22] **13n**: [43]). See Supplementary Materials for copies of NMR spectra. 

#### 3.2.1. 2-Phenylbenzofuran (**13a**)

Colorless plates (87.3 mg, 90%). mp 111–113 °C (from hexane). ^1^H-NMR δ: 7.88 (d, *J* = 7.8 Hz, 2H, Ar-H), 7.60 (d, *J* = 7.3 Hz, 1H, Ar-H), 7.54 (d, *J* = 8.2 Hz, 1H, Ar-H), 7.46 (t, *J* = 7.3 Hz, 2H, Ar-H), 7.36 (t, *J* = 7.8 Hz, 1H, Ar-H), 7.30 (td, *J* = 7.3, 1.4 Hz, 1H, Ar-H), 7.24 (t, *J* = 7.3 Hz, 1H, Ar-H), 7.04 (s, 1H, Ar-H). ^13^C-NMR δ: 156.0 (C), 155.0 (C), 130.6 (C), 129.3 (C), 128.9 (CH), 128.7 (CH), 125.0 (CH), 124.4 (CH), 123.0 (CH), 121.0 (CH), 111.3 (CH), 101.4 (CH). LRMS (EI) *m/z*: 194 ([M]^+^, 100%), 165 (50%), 130 (5%).

#### 3.2.2. 2-(4-Methoxyphenyl) Benzofuran (**13b**)

Colorless plates (67.2 mg, 60%). mp 146–148 °C (from hexane). ^1^H-NMR δ: 7.80 (dt, *J* = 9.6, 2.7 Hz, 2H, Ar-H), 7.55 (dd, *J* = 7.3, 1.8 Hz, 1H, Ar-H), 7.50 (d, *J* = 7.8 Hz, 1H, Ar-H), 7.27–7.19 (m, 2H, Ar-H), 6.98 (dt, *J* = 9.6, 2.7 Hz, 2H, Ar-H), 6.89 (s, 1H, Ar-H), 3.87 (s, 3H, OCH_3_). ^13^C-NMR δ: 160.1 (C), 156.1 (C), 154.8 (C), 129.6 (C), 126.5 (CH), 123.8 (CH), 123.4 (C), 122.9 (CH), 120.7 (CH), 114.3 (CH), 111.1 (CH), 99.8 (CH), 55.5 (OCH_3_). LRMS (EI) *m/z*: 224 ([M]^+^, 100%), 181 (38%), 152 (22%), 112 (7%).

#### 3.2.3. 2-(*p*-Tolyl) Benzofuran (**13c**)

Colorless plates (77.0 mg, 74%). mp 128–130 °C (from hexane). ^1^H-NMR δ: 7.76 (d, *J* = 8.2 Hz, 2H, Ar-H), 7.57 (dd, *J* = 6.4, 0.9 Hz, 1H, Ar-H), 7.51 (dd, *J* = 8.2, 0.9 Hz, 1H, Ar-H), 7.29–7.20 (m, 4H, Ar-H), 6.97 (s, 1H, Ar-H), 2.40 (s, 3H, CH_3_). ^13^C-NMR δ: 156.2 (C), 154.7 (C), 138.6 (C), 129.5 (CH), 129.3 (C), 127.7 (C), 124.9 (CH), 124.0 (CH), 122.8 (CH), 120.7 (CH), 111.1 (CH), 100.5 (CH), 21.4 (CH_3_). LRMS (EI) *m/z*: 208 ([M]^+^, 100%), 178 (18%), 152 (7%), 115 (4%), 89 (9%), 63 (4%).

#### 3.2.4. 2-(4-Chlorophenyl) Benzofuran (**13d**)

Colorless plates (92.6 mg, 81%). mp 142–144 °C (from hexane). ^1^H-NMR δ: 7.78 (dt, *J* = 9.1, 2.7 Hz, 2H, Ar-H), 7.58 (d, *J* = 7.3 Hz, 1H, Ar-H), 7.51 (d, *J* = 8.2 Hz, 1H, Ar-H), 7.41 (dt, *J* = 9.1, 2.7 Hz, 2H, Ar-H), 7.29 (td, *J* = 7.3, 1.4 Hz, 1H, Ar-H), 7.25–7.21 (m, 1H, Ar-H), 7.00 (d, *J* = 0.9 Hz, 1H, Ar-H). ^13^C-NMR δ: 155.0 (C), 154.9 (C), 134.4 (C), 129.13 (CH), 129.06 (C), 126.2 (CH), 124.7 (CH), 123.2 (CH), 121.1 (CH), 111.3 (CH), 101.8 (CH). LRMS (EI) *m/z*: 228 ([M]^+^, 100%), 199 (6%), 165 (37%), 139 (4%), 114 (8%), 82 (7%).

#### 3.2.5. 2-(4-Bromophenyl) Benzofuran (**13e**)

Colorless plates (124.3 mg, 91%). mp 162–164 °C (from hexane). ^1^H-NMR δ: 7.73 (dt, *J* = 9.1, 2.3 Hz, 2H, Ar-H), 7.60–7.56 (m, 3H, Ar-H), 7.52 (d, *J* = 8.2 Hz, 1H, Ar-H), 7.30 (td, *J* = 7.3, 1.4 Hz, 1H, Ar-H), 7.24 (td, *J* = 7.8, 1.4 Hz, 1H, Ar-H), 7.03 (d, *J* = 0.9 Hz, 1H, Ar-H). ^13^C-NMR δ: 154.9 (C), 154.7 (C), 131.9 (CH), 129.4 (C), 129.0 (C), 126.3 (CH), 124.6 (CH), 123.1 (CH), 122.5 (C), 121.0 (CH), 111.2 (CH), 101.8 (CH). LRMS (EI) *m/z*: 272 ([M]^+^, 100%), 165 (68%), 137 (11%), 115 (5%), 83 (15%), 63 (6%).

#### 3.2.6. Ethyl 4-(benzofuran-2-yl) Benzoate (**13f**)

Colorless plates (71.6 mg, 53%). mp 142–144 °C (from hexane). ^1^H-NMR δ: 8.12 (dt, *J* = 8.7, 1.8 Hz, 2H, Ar-H), 7.92 (dt, *J* = 8.7, 1.8 Hz, 2H, Ar-H), 7.61 (dd, *J* = 8.2, 0.9 Hz, 1H, Ar-H), 7.54 (d, *J* = 8.2 Hz, 1H, Ar-H), 7.33 (td, *J* = 7.3, 1.4 Hz, 1H, Ar-H), 7.25 (td, *J* = 5.9, 0.9 Hz, 1H, Ar-H), 7.15 (s, 1H, Ar-H), 4.41 (q, *J* = 7.3 Hz, 2H, CH_2_), 1.42 (t, *J* = 7.3 Hz, 3H, CH_3_). ^13^C-NMR δ: 166.2 (C), 155.1 (C), 154.6 (C), 134.3 (C), 130.1 (CH), 130.0 (C), 128.9 (C), 125.0 (CH), 124.5 (CH), 123.2 (CH), 121.2 (CH), 111.3 (CH), 103.3 (CH), 61.1 (CH_2_), 14.3 (CH_3_). LRMS (EI) *m/z*: 266 ([M]^+^, 100%), 221 (67%), 193 (12%), 165 (52%), 139 (10%), 111 (7%), 83 (10%). IR (KBr): 2976, 1709, 1611, 1273, 1099, 748 cm^−1^.

#### 3.2.7. 2-[4-(Trifluoromethyl)phenyl] Benzofuran (**13g**)

Colorless needles (48.5 mg, 37%). mp 161–163 °C (from hexane). ^1^H-NMR δ: 7.97 (d, *J* = 7.8 Hz, 2H, Ar-H), 7.70 (d, *J* = 8.2 Hz, 2H, Ar-H), 7.62 (dd, *J* = 6.9, 0.9 Hz, 1H, Ar-H), 7.54 (dd, *J* = 7.3, 0.9 Hz, 1H, Ar-H), 7.34 (td, *J* = 7.3, 1.4 Hz, 1H, Ar-H), 7.26 (td, *J* = 7.8, 1.4 Hz, 1H, Ar-H), 7.15 (s, 1H, Ar-H). ^13^C-NMR δ: 155.2 (C), 154.3 (C), 133.8 (C), 130.2 (q, ^2^*J*_C,F_ = 33 Hz, C), 128.9 (C), 125.9 (q, ^3^*J*_C,F_ = 2.9 Hz, CH), 125.2 (CH), 125.0 (CH), 124.2 (q, ^1^*J*_C,F_ = 276 Hz, C), 123.4 (CH), 121.4 (CH), 111.5 (CH), 103.3 (CH). ^19^F-NMR δ: −62.5. LRMS (EI) *m/z*: 262 ([M]^+^, 100%), 233 (12%), 183 (6%), 165 (30%), 131 (4%), 106 (8%).

#### 3.2.8. 2-(*o*-Tolyl) Benzofuran (**13i**)

Pale yellow oil (63.5 mg, 61%). ^1^H-NMR δ: 7.88–7.85 (m, 1H, Ar-H), 7.62 (dd, *J* = 7.3, 1.4 Hz, 1H, Ar-H), 7.54 (dd, *J* = 8.2, 0.9 Hz, 1H, Ar-H), 7.33–7.29 (m, 4H, Ar-H), 7.25 (td, *J* = 7.8, 1.4 Hz, 1H, Ar-H), 6.91 (s, 1H, Ar-H), 2.60 (s, 3 H, CH_3_). ^13^C-NMR δ: 155.7 (C), 154.5 (C), 135.9 (C), 131.4 (CH), 130.0 (C), 129.3 (C), 128.6 (CH), 128.2 (CH), 126.2 (CH), 124.3 (CH), 122.9 (CH), 121.0 (CH), 111.2 (CH), 105.2 (CH), 22.1 (CH_3_). LRMS (EI) *m/z*: 208 ([M]^+^, 100%), 178 (33%), 165 (14%), 152 (12%), 115 (14%), 89 (19%).

#### 3.2.9. 5-Methoxy-2-phenylbanzofuran (**13j**)

Colorless needles (62.8 mg, 56%). mp 128–130 °C (from hexane). ^1^H-NMR δ: 7.84–7.81 (m, 2H, Ar-H), 7.45–7.39 (m, 3H, Ar-H), 7.35–7.31 (m, 1H, Ar-H), 7.02 (d, *J* = 2.7 Hz, 1H, Ar-H), 6.94 (d, *J* = 0.9 Hz, 1H, Ar-H), 6.88 (dd, *J* = 9.1, 2.7 Hz, 1H, Ar-H), 3.84 (s, 3H, OCH_3_). ^13^C-NMR δ: 156.8 (C), 156.1 (C), 150.0 (C), 130.6 (C), 129.9 (C), 128.9 (CH), 128.6 (CH), 124.9 (CH), 113.1 (CH), 111.7 (CH), 103.4 (CH), 56.0 (OCH_3_). LRMS (EI) *m/z*: 224 ([M]^+^, 100%), 181 (18%), 152 (33%), 127 (7%).

#### 3.2.10. 5-Methyl-2-phenylbanzofuran (**13k**)

Colorless needles (79.1 mg, 76%). mp 128–130 °C (from hexane). ^1^H-NMR δ: 7.85–7.83 (m, 2H, Ar-H), 7.44–7.38 (m, 3H, Ar-H), 7.34–7.30 (m, 2H, Ar-H), 7.07 (dd, *J* = 8.2, 1.4 Hz, 1H, Ar-H), 6.92 (d, *J* = 0.9 Hz, 1H, Ar-H), 2.43 (s, 3H, CH_3_). ^13^C-NMR δ: 156.1 (C), 153.4 (C), 132.4 (C), 130.7 (C), 129.4 (C), 128.9 (CH), 128.5 (CH), 125.7 (CH), 125.0 (CH), 120.9 (CH), 110.8 (CH), 101.2 (CH), 21.5 (CH_3_). LRMS (EI) *m/z*: 208 ([M]^+^, 100%), 178 (20%), 152 (7%), 104 (9%), 76 (7%).

#### 3.2.11. 5-Chloro-2-phenylbanzofuran (**13l**)

Colorless plates (112.0 mg, 98%). mp 154–156 °C (from hexane). ^1^H-NMR δ: 7.84 (dt, *J* = 8.2, 1.4 Hz, 2H, Ar-H), 7.53 (d, *J* = 2.3 Hz, 1H, Ar-H), 7.47–7.41 (m, 3H, Ar-H), 7.39–7.35 (m, 1H, Ar-H), 7.22 (dd, *J* = 8.7, 1.8 Hz, 1H, Ar-H), 6.95 (s, 1H, Ar-H). ^13^C-NMR δ: 157.5 (C), 153.3 (C), 130.7 (C), 130.0 (C), 129.1 (CH), 129.0 (CH), 128.6 (C), 125.1 (CH), 124.5 (CH), 120.5 (CH), 112.2 (CH), 100.9 (CH). LRMS (EI) *m/z*: 228 ([M]^+^, 100%), 165 (40%), 139 (9%), 114 (9%), 82 (10%).

#### 3.2.12. 5-Bromo-2-phenylbanzofuran (**13m**)

Colorless needles (112.2 mg, 82%). mp 159–162 °C (from hexane). ^1^H-NMR δ: 7.85–7.82 (m, 2H, Ar-H), 7.69 (dd, *J* = 1.8, 0.9 Hz, 1H, Ar-H), 7.47–7.42 (m, 2H, Ar-H), 7.40–7.34 (m, 3H, Ar-H), 6.94 (s, 1H, Ar-H). ^13^C-NMR δ: 157.3 (C), 153.7 (C), 131.3 (C), 130.0 (C), 129.1 (CH), 129.0 (CH), 127.2 (CH), 125.2 (CH), 123.6 (CH), 116.1 (C), 112.7 (CH), 100.7 (CH). LRMS (EI) *m/z*: 272 ([M]^+^, 100%), 193 (10%), 165 (50%), 139 (21%), 115 (6%), 82 (18%), 63 (7%).

#### 3.2.13. 2-Phenylbanzofuran-5-carbonitrile (**13n**)

Colorless needles (86.6 mg, 79%). mp 143–145 °C (from hexane). ^1^H-NMR δ: 7.91 (d, *J* = 0.9 Hz, 1H, Ar-H), 7.87–7.84 (m, 2H, Ar-H), 7.59 (d, *J* = 8.2 Hz, 1H, Ar-H), 7.55 (dd, *J* = 8.2, 1.4 Hz, 1H, Ar-H), 7.50–7.46 (m, 2H, Ar-H), 7.43–7.39 (m, 1H, Ar-H), 7.05 (d, *J* = 0.9 Hz, 1H, Ar-H). ^13^C-NMR δ: 158.4 (C), 156.5 (C), 130.0 (C), 129.7 (CH), 129.3 (C), 129.1 (CH), 128.0 (CH), 125.8 (CH), 125.3 (CH), 119.6 (C), 112.4 (CH), 106.9 (C), 100.8 (CH). LRMS (EI) *m/z*: 219 ([M]^+^, 100%), 190 (36%), 164 (7%), 110 (7%), 82 (20%). FTIR (KBr): 2226, 1792, 1541, 826, 419 cm^−1^.

### 3.3. Crystal Structure Determination

Single crystals were obtained from dichloromethane/hexane. A suitable crystal was selected on a XtaLAB Synergy, Dualflex, HyPix diffractometer (Rigaku, Tokyo, Japan). The crystal was kept at 100 K during data collection. Using Olex2 [44], the structure was solved with the ShelXT [45] structure solution program using Intrinsic Phasing and refined with the ShelXL [46] refinement package using Least Squares minimisation.

#### Crystal Data for **2c**

C_21_H_21_F_2_Sb (*M* = 433.13 g/mol): monoclinic, space group *C*2/*c* (No. 15), *a* = 26.6218(3) Å, *b* = 10.43710(10) Å, *c* = 21.8927(2) Å, β = 113.3610(10)°, *V* = 5584.33(10) Å^3^, *Z* = 12, *T* = 100 K, *μ*(CuKα) = 11.904 mm^−1^, *D_calc_* = 1.546 g/cm^3^, 36,807 reflections measured (7.234° ≤ 2*Θ* ≤ 153.916°), 5816 unique (*R*_int_ = 0.0305, *R_sigma_* = 0.0193) which were used in all calculations. The final *R*_1_ was 0.0179 (I > 2σ(I)) and *wR*_2_ was 0.0458 (all data). CCDC #2036952.

## 4. Conclusions

Pd-catalyzed regioselective C–H arylation of benzofurans with Ar_3_SbF_2_ proceeded under aerobic conditions to afford 2-arylbenzofuran derivatives. The reaction of Ar_3_SbF_2_ bearing various functional groups afforded the corresponding coupling products under mild conditions, with the exception of electron-withdrawing groups, such as nitrile, on the phenyl ring. Furthermore, the reaction of 5-substituted benzofurans with Ph_3_SbF_2_ also proceeded smoothly. X-ray crystallography of *p*-Tol_3_SbF_2_ revealed that antimony is the central atom of a trigonal bipyramidal structure, and intramolecular hydrogen bonds of the C–H∙∙∙F type exist between the fluorines and the hydrogens at the *ortho* positions on the phenyl rings. Detailed mechanistic studies of this cross-coupling and the reactions of Ar_3_SbF_2_ with other coupling partners are underway.

## Figures and Tables

**Table 1:**
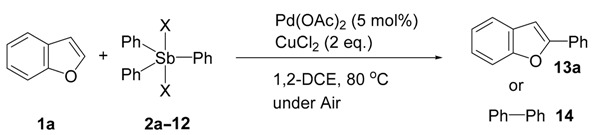
Pd-catalyzed reaction of benzofuran **1a** with pentavalent organoantimony compounds **2a**–**12** ^a^.

Entry	Sb Reagent	Time (h)	Yield (%) ^b^	
			13a	14
1	Ph_3_SbF_2_ **2a**	3	93 (90) ^c^	---
2	Ph_3_SbCl_2_ **3**	6	88	3
3	Ph_3_SbBr_2_ **4**	24	2	<1
4	Ph_3_Sb(OAc)_2_ **5**	3	62	37
5	Ph_3_Sb(OCOCF_3_)_2_ **6**	6	72	23
6	Ph_3_Sb(OBz)_2_ **7**	6	70	24
7	Ph_3_Sb(OTs)_2_ **8**	6	79	---
8	Ph_3_Sb(OCHO)_2_ **9**	6	58	11
9	Ph_4_SbBr **10**	6	47	7 ^d^
10	Ph_4_SbOAc **11**	6	63	25 ^d^
11	Ph_5_Sb **12**	24	77	22 ^e^

^a^ Condition: **1a** (0.5 mmol), **2a**–**12** (0.5 mmol), Pd(OAc)_2_ (0.025 mmol), CuCl_2_ (1 mmol). ^b^ GC yield using dibenzyl as internal standard. The yield 100% corresponds to the formation of 0.5 mmol of **13** and 0.75 mmol of **14**. ^c^ Isolated yield. ^d^ Yield 100% corresponds to the formation of 1 mmol of **14**. ^e^ Yield 100% corresponds to the formation of 1.25 mmol of **14**.

**Table 2:**
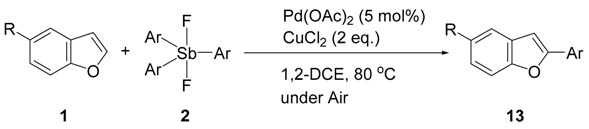
Pd-catalyzed reaction of benzofurans with Ar_3_SbF_2_^a,b^.

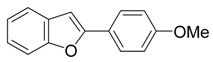	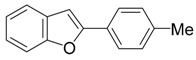	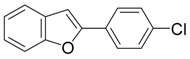
**13b**: 19% (1 h) **13b**^c^: 60% (3 h)	**13c**: 74% (1.5 h)	**13d**: 81% (4 h)
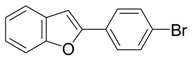	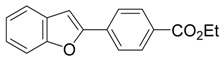	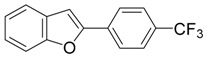
**13e**: 91% (5 h)	**13f**: 53% (24 h)	**13g**: 37% (72 h)
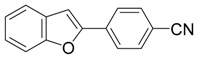	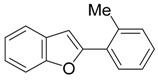	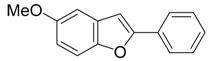
**13h**: 0% (72 h)	**13i**: 61% (24 h)	**13j**: 56% (24 h)
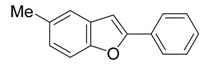	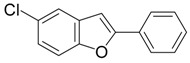	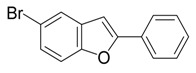
**13k**: 76% (5 h)	**13l**: 98% (24 h)	**13m**: 82% (24 h)
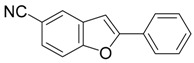	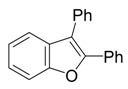	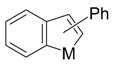
**13n**: 79% (24 h)	**13o**^d^: 0% (24 h)	**13p** (M = Se): 0% (24 h) **13q** (M = Te): 0% (24 h)

^a^ Conditions: **1** (0.5 mmol), **2** (0.5 mmol), Pd(OAc)_2_ (0.025 mmol), CuCl_2_ (1 mmol). ^b^ Isolated yields. ^c^ 40 °C. ^d^ **2a** (1 mmol).

**Table 3:** Selected bond lengths (Å) and bond angles (°) for *p*-Tol_3_SbF_2_ **2c**.

	*p*-Tol_3_SbF_2_		
Bond lengths (Å)		Atomic distance (Å)	
Sb–F(1)	1.9839(10)	H(2)…F(1)	2.30(3)
Sb–F(2)	1.9843(10)	H(5)…F(1)	2.43(3)
Sb–C(1)	2.1021(17)	H(8)…F(1)	2.49(2)
Sb–C(4)	2.1017(16)	H(3)…F(2)	2.34(3)
Sb–C(7)	2.1012(16)	H(6)…F(2)	2.28(3)
Bond angles (°)		H(9)…F(2)	2.50(2)
F(1)–Sb–F(2)	178.93(4)	Dihedral angles (°)	
C(1)–Sb–C(4)	123.62(6)	C(2)–C(1)–Sb–F(1)	−8.58(13)
C(1)–Sb–C(7)	118.16(6)	C(5)–C(4)–Sb–F(1)	−10.80(18)
C(4)–Sb–C(7)	118.22(6)	C(8)–C(7)–Sb–F(1)	23.45(14)
F(1)–Sb–C(1)	89.19(6)	C(3)–C(1)–Sb–F(2)	−6.71(13)
F(1)–Sb–C(4)	90.36(6)	C(6)–C(4)–Sb–F(2)	−14.45(18)
F(1)–Sb–C(7)	90.83(5)	C(9)–C(7)–Sb–F(2)	25.99(15)
F(2)–Sb–C(1)	89.98(6)		
F(2)–Sb–C(4)	89.53(6)		
F(2)–Sb–C(7)	90.16(5)		

## Supplementary Materials

The following are available online, Figures S1–S13: ^1^H-NMR and ^13^C-NMR spectra of the products. Cif files are also provided as supplementary.

## Sample Availability

Samples of the organoantimony compounds are available from the authors.
