# Supplementary material for: Palladium-Catalyzed C–H Arylation of Benzofurans with Triarylantimony Difluorides for the Synthesis of 2-Arylbenzofurans
Source: Molecules. 2020 Dec 28;26(1):97. doi: 10.3390/molecules26010097 (PMC7795347; doi:10.3390/molecules26010097)

Figure S1. NMR spectra of **13a**.

^{1}H-NMR of **13a**

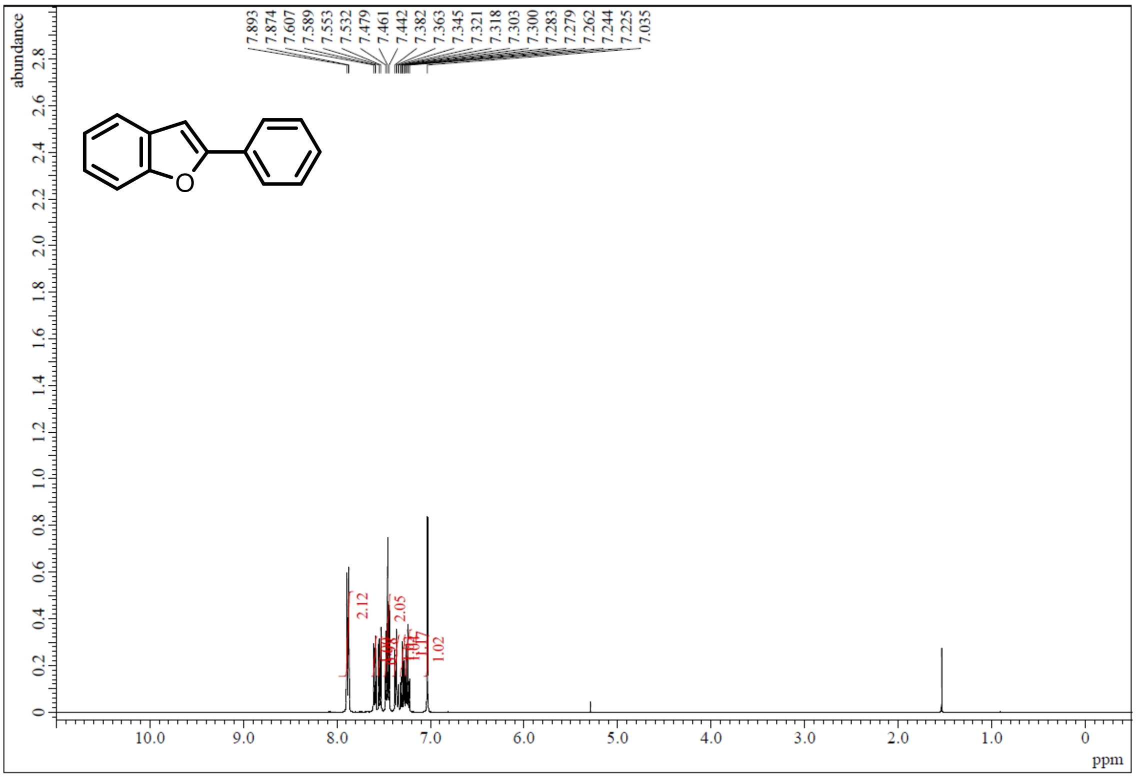

^{13}C-NMR of **13a**

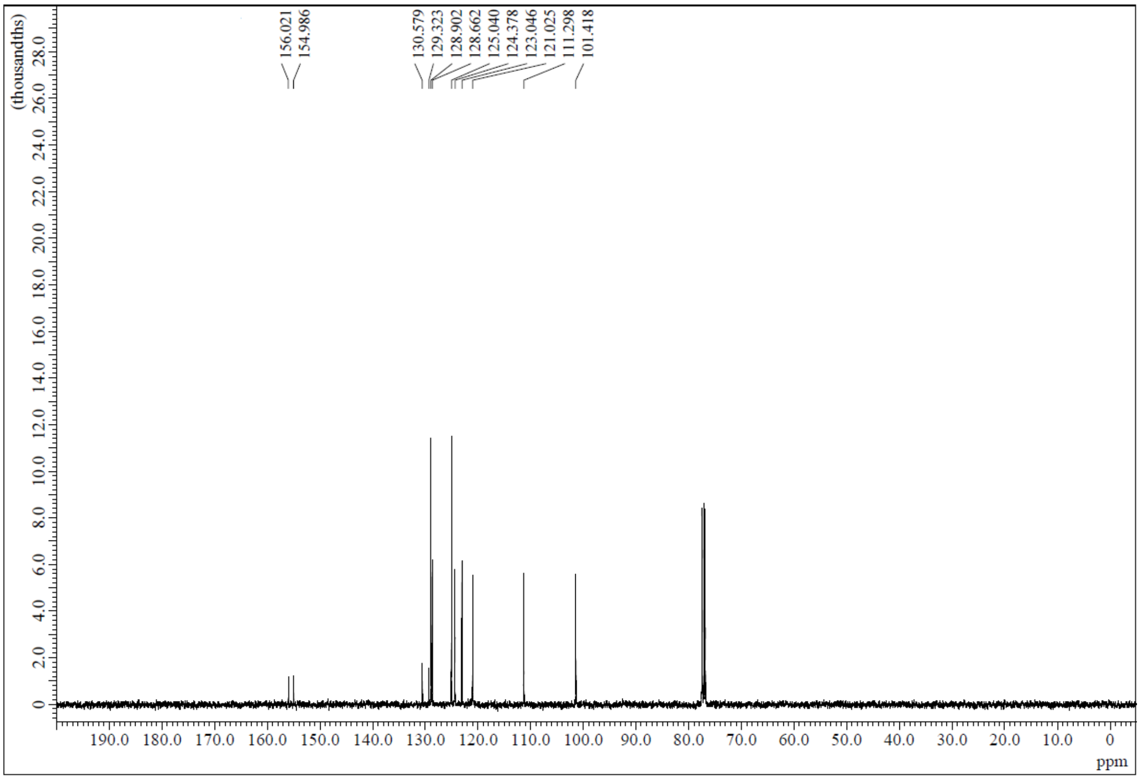

Figure S2. NMR spectra of **13b**.

^{1}H-NMR of **13b**

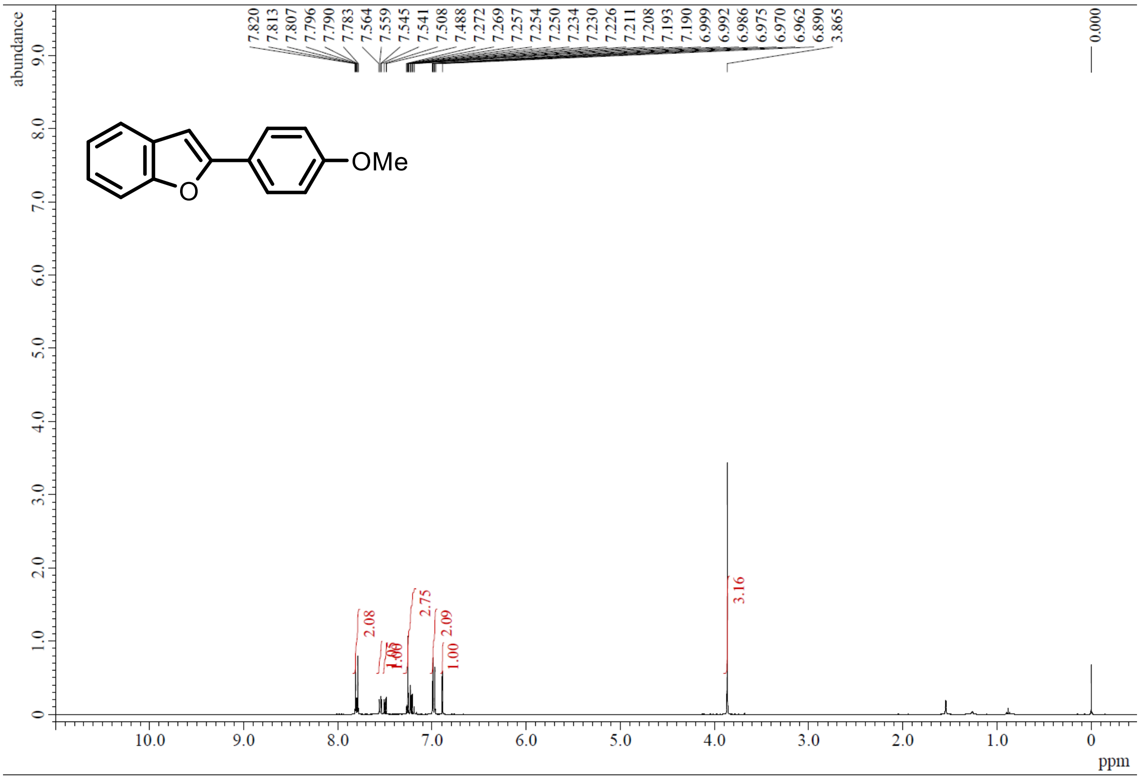

^{13}C-NMR of **13b**

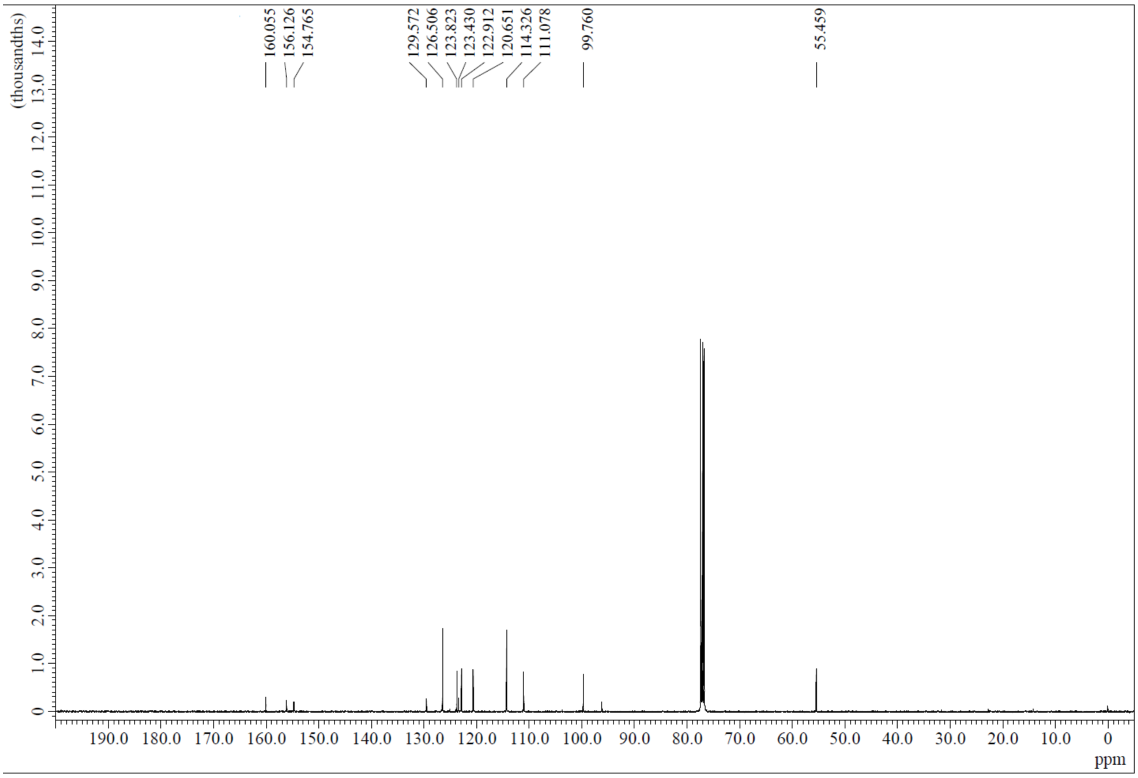

Figure S3. NMR spectra of **13c**.

^{1}H-NMR of **13c**

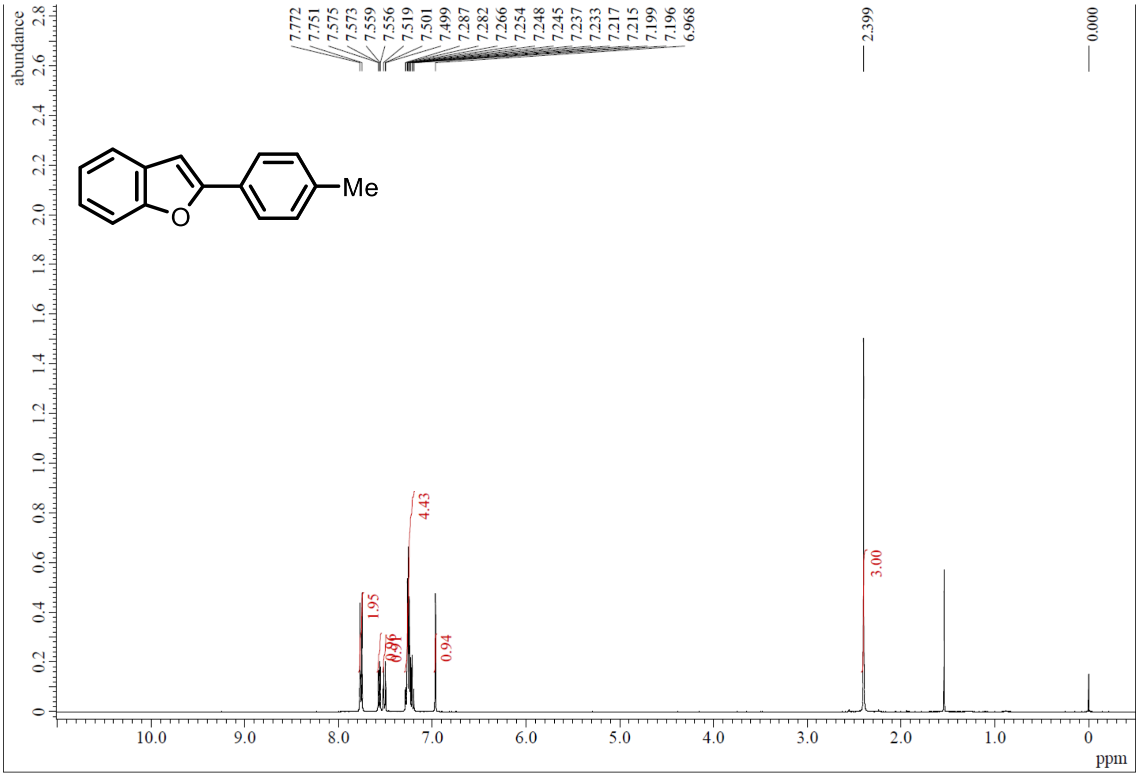

^{13}C-NMR of **13c**

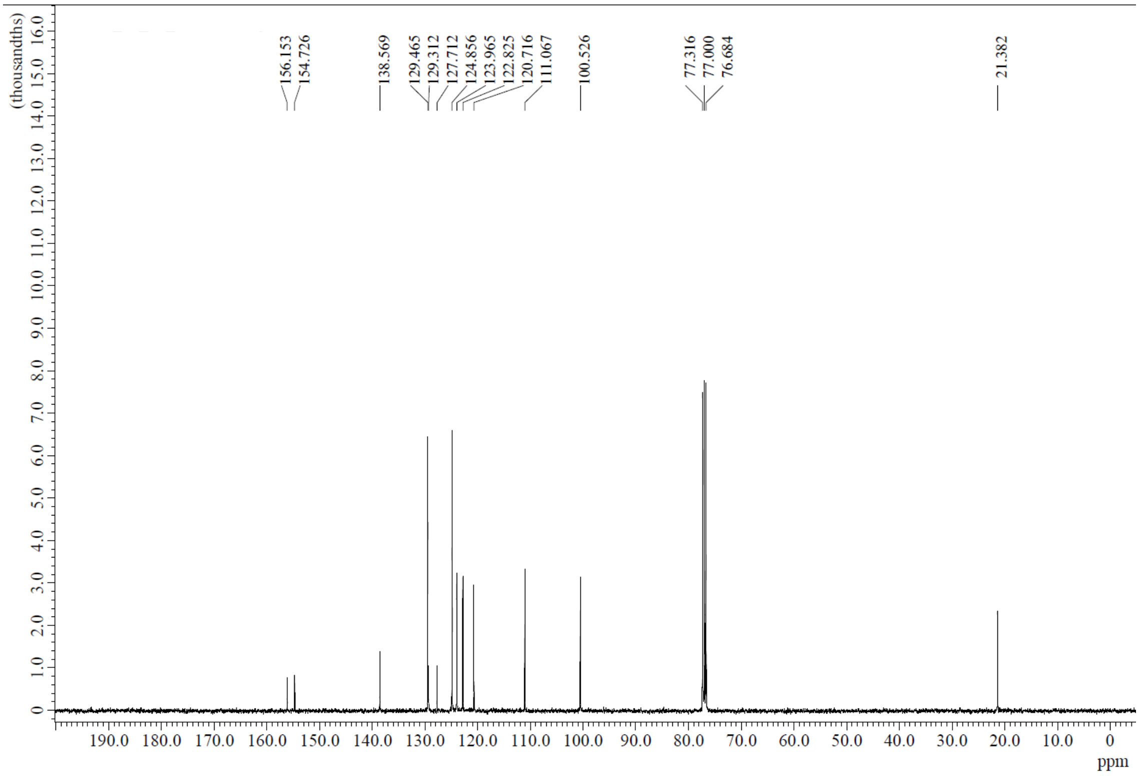

Figure S4. NMR spectra of **13d**.

^{1}H-NMR of **13d**

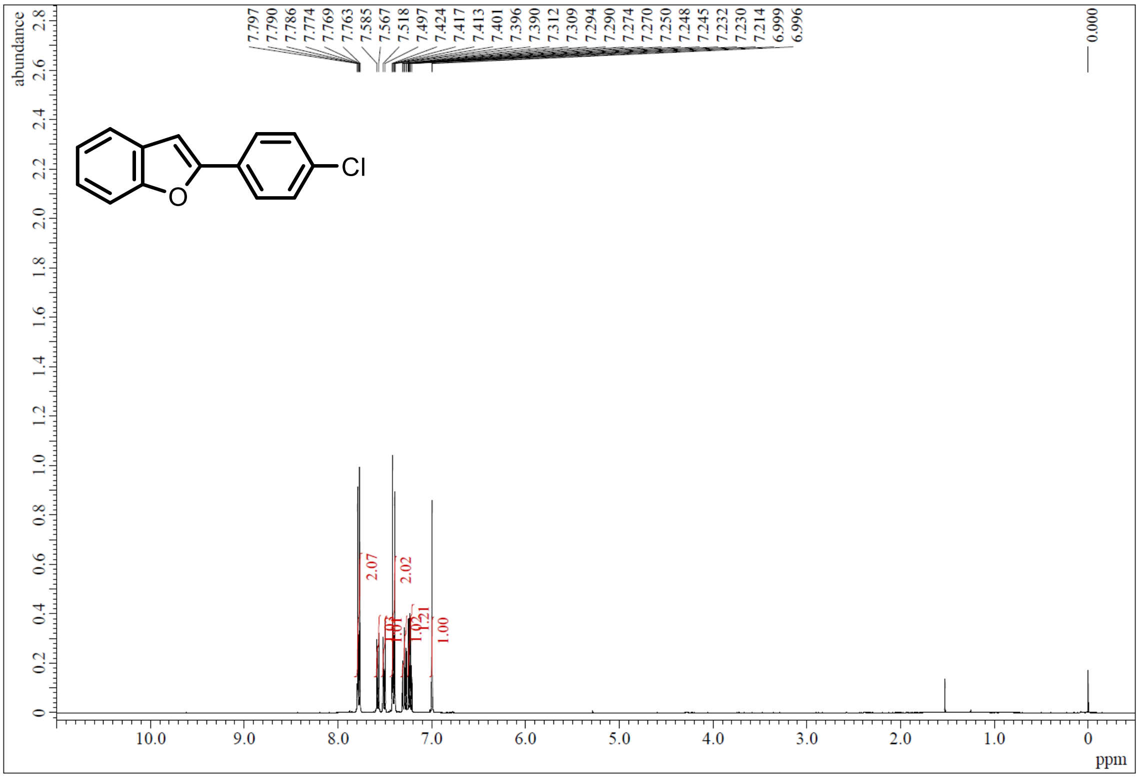

^{13}C-NMR of **13d**

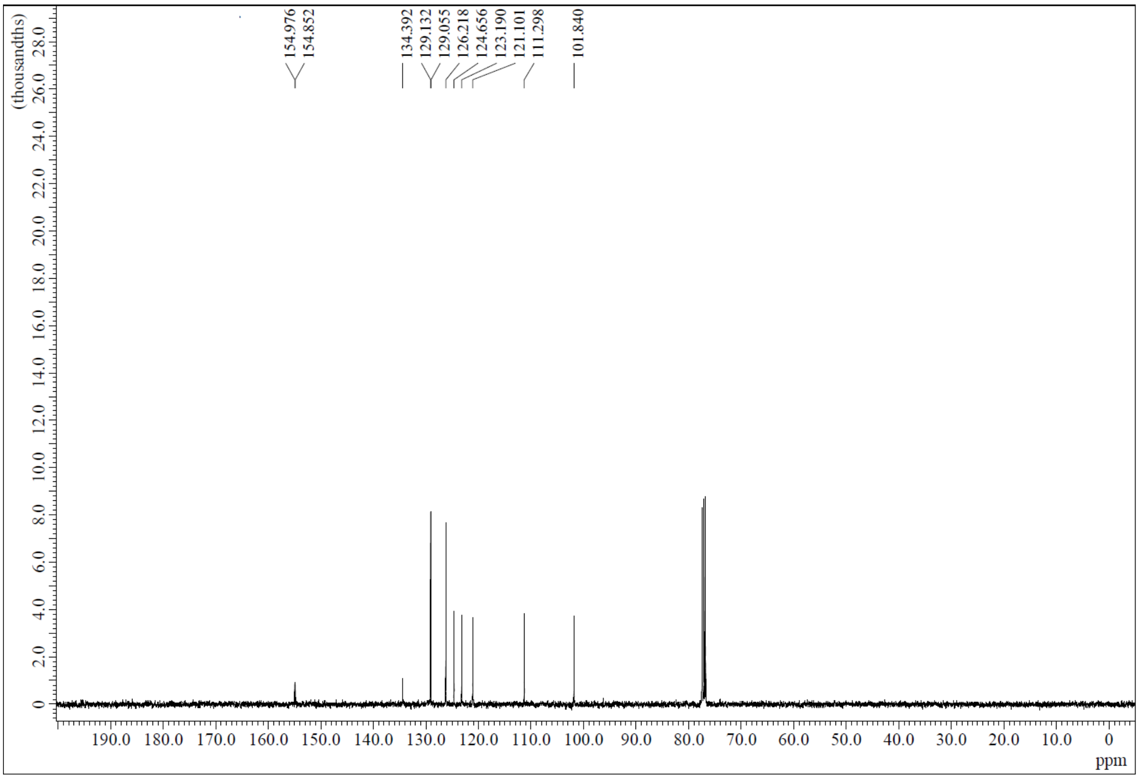

Figure S5. NMR spectra of **13e**.

^{1}H-NMR of **13e**

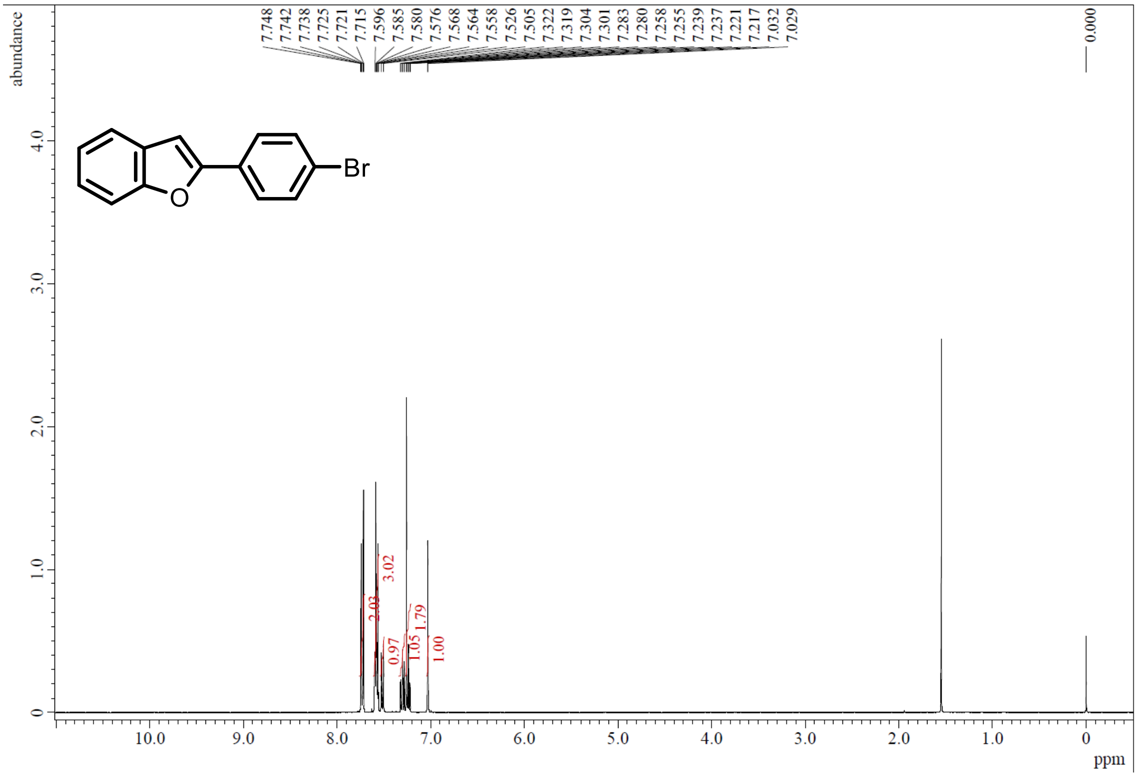

^{13}C-NMR of **13e**

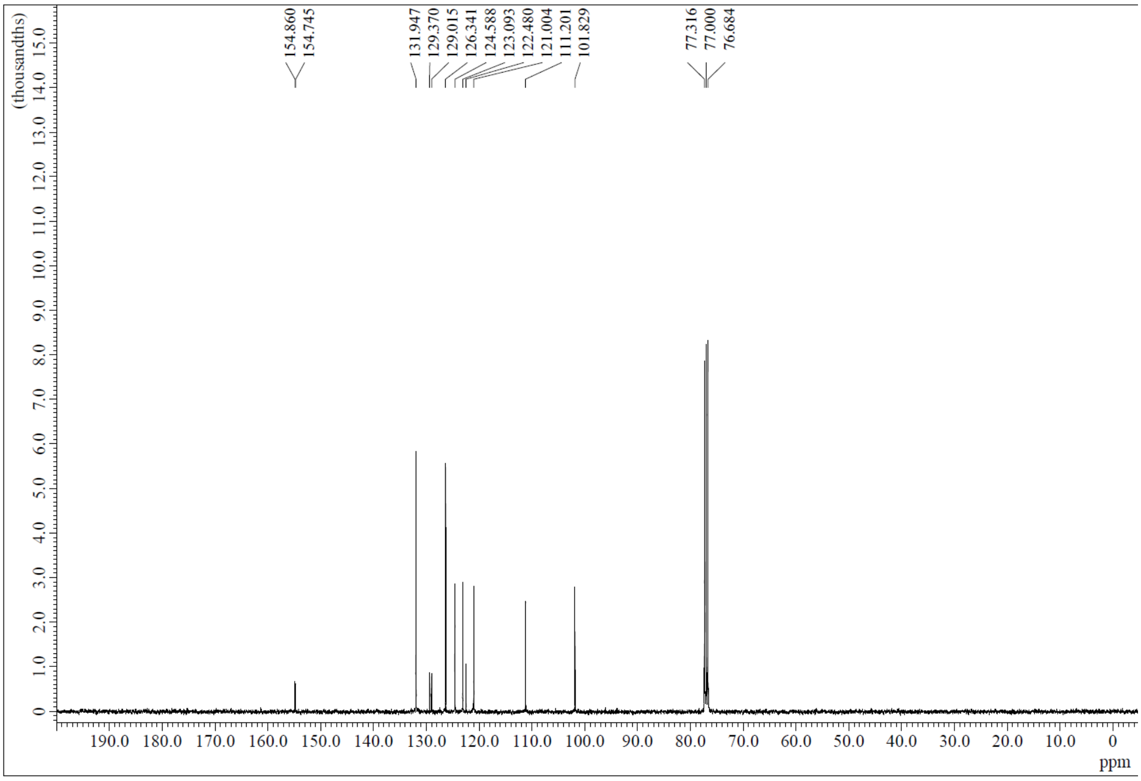

Figure S6. NMR spectra of **13f**.

^{1}H-NMR of **13f**

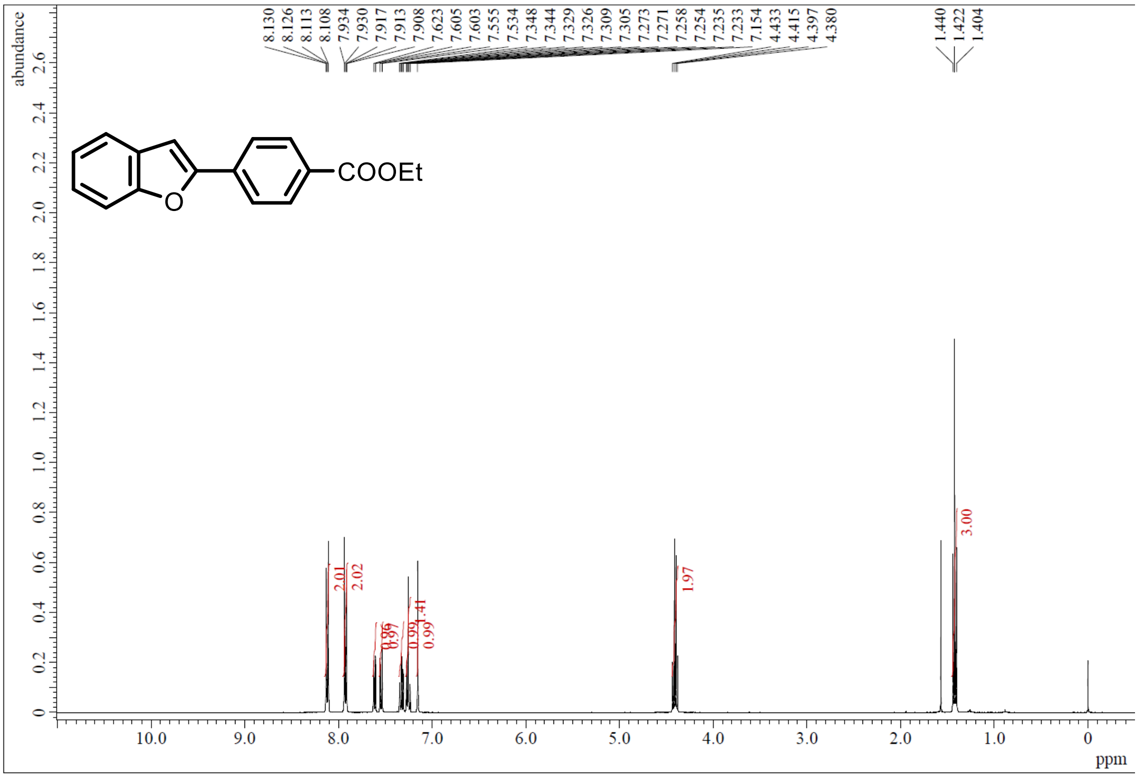

^{13}C-NMR of **13f**

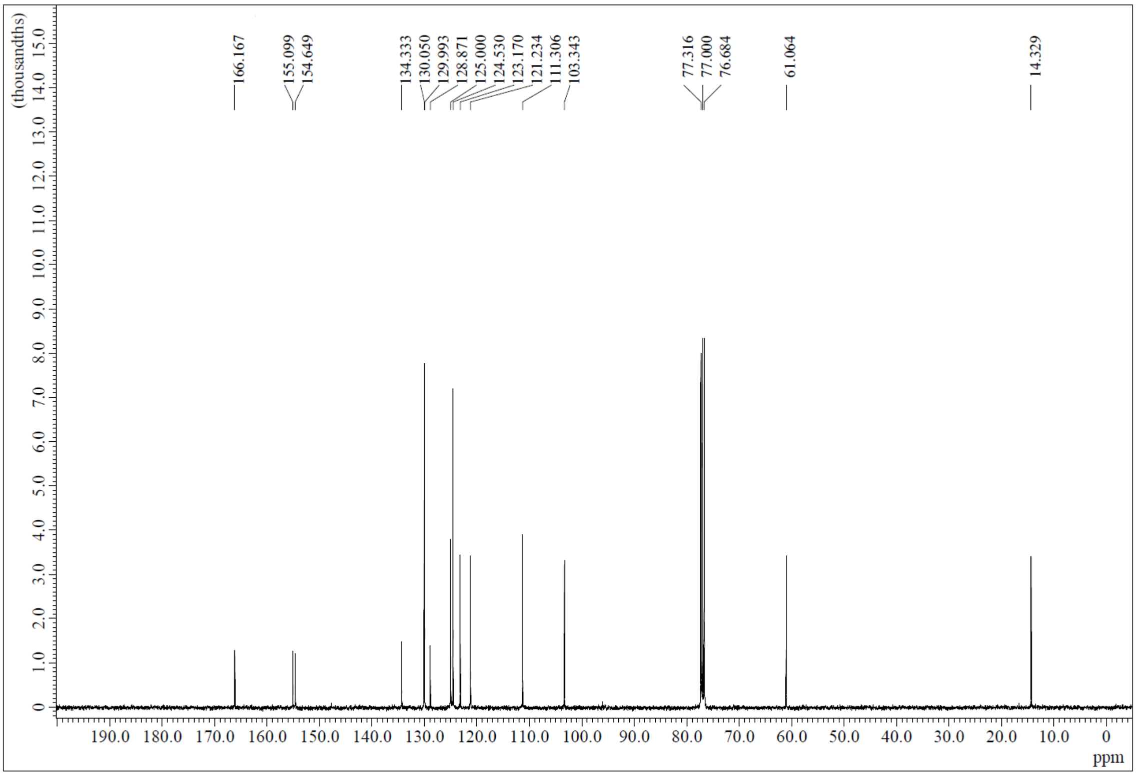

Figure S7. NMR spectra of **13g**.

^{1}H-NMR of **13g**

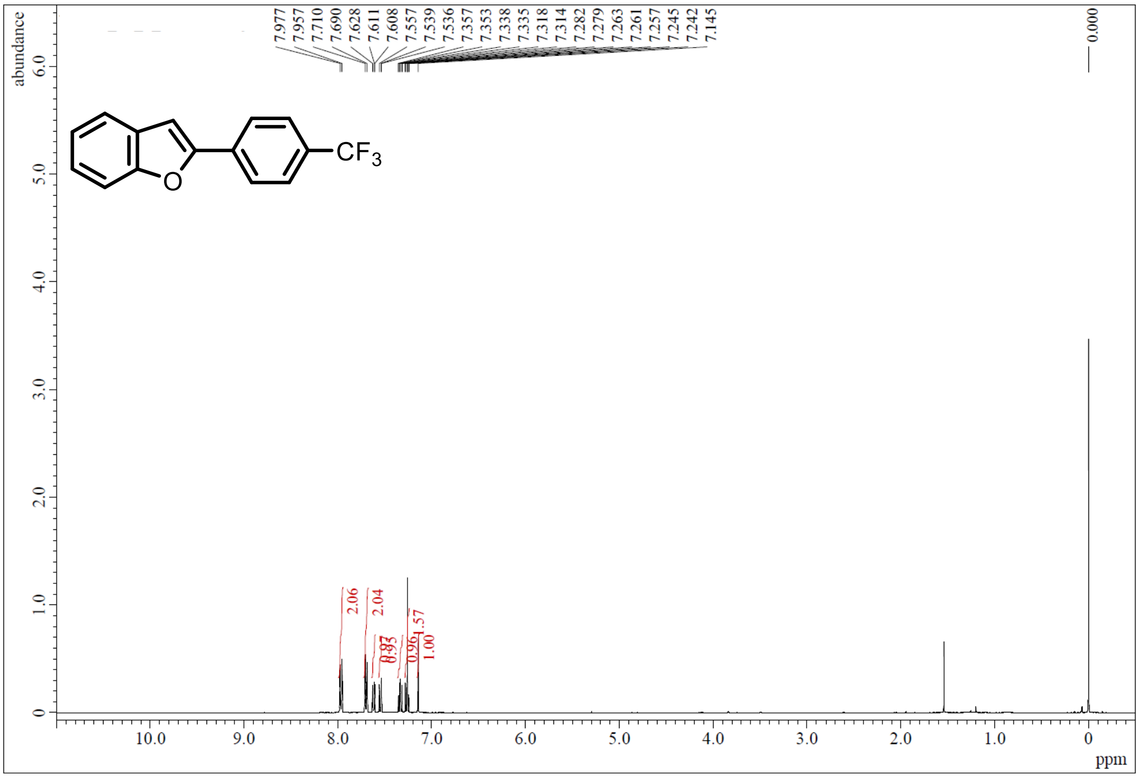

^{13}C-NMR of **13g**

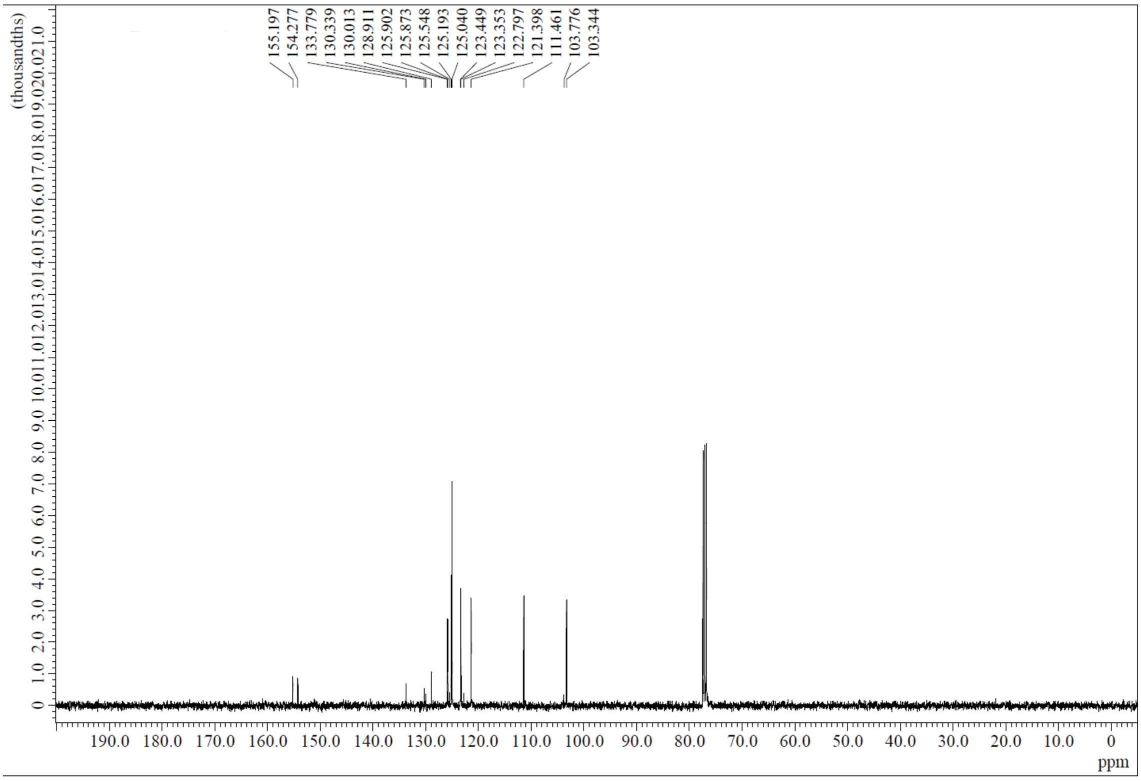

Figure S8. NMR spectra of **13i**.

^{1}H-NMR of **13i**

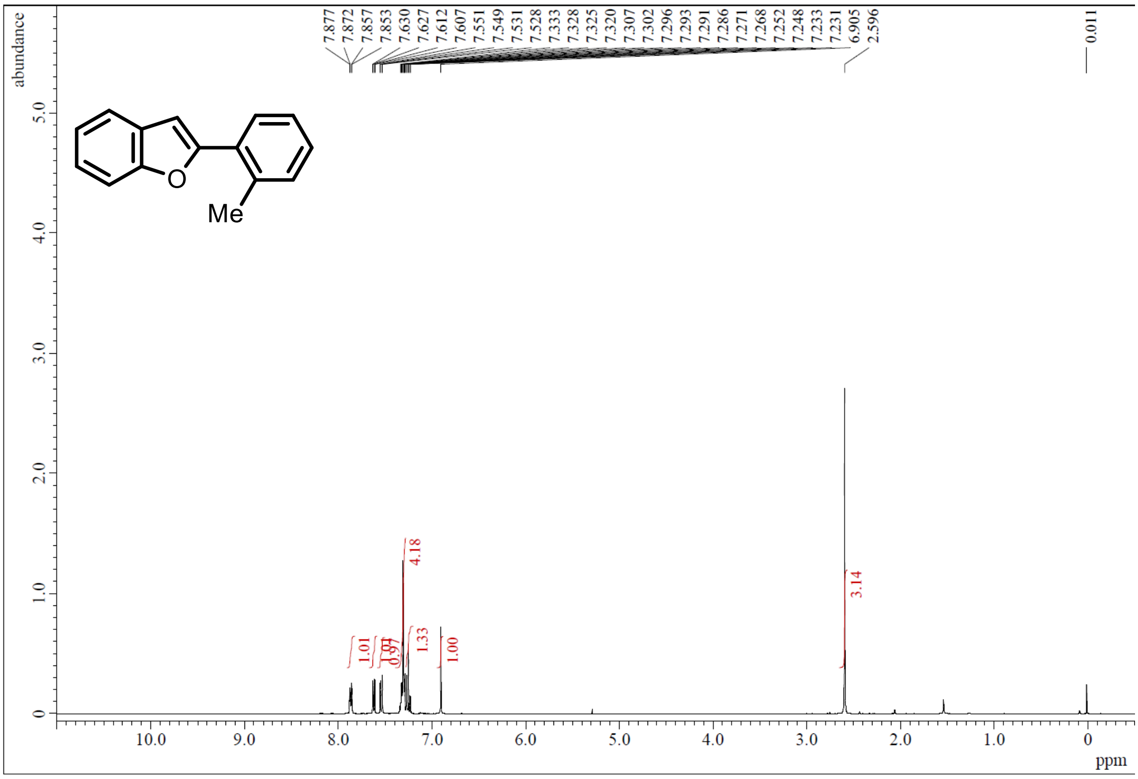

^{13}C-NMR of **13i**

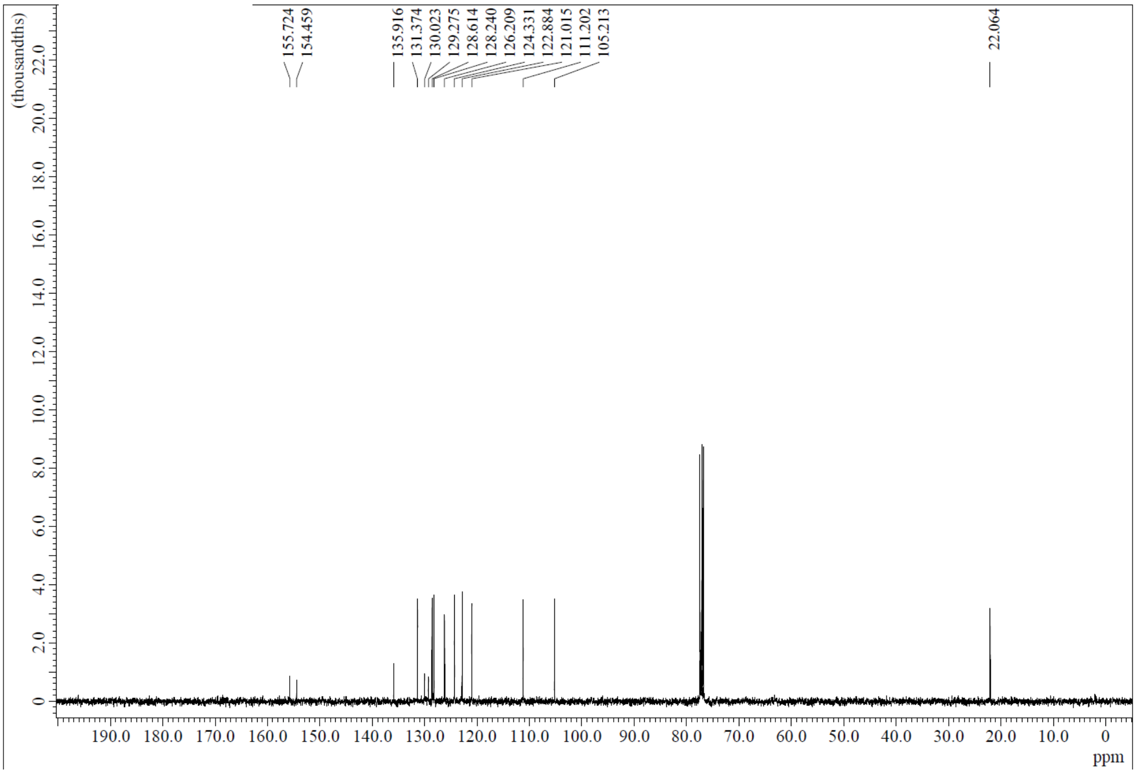

Figure S9. NMR spectra of **13j**.

^{1}H-NMR of **13j**

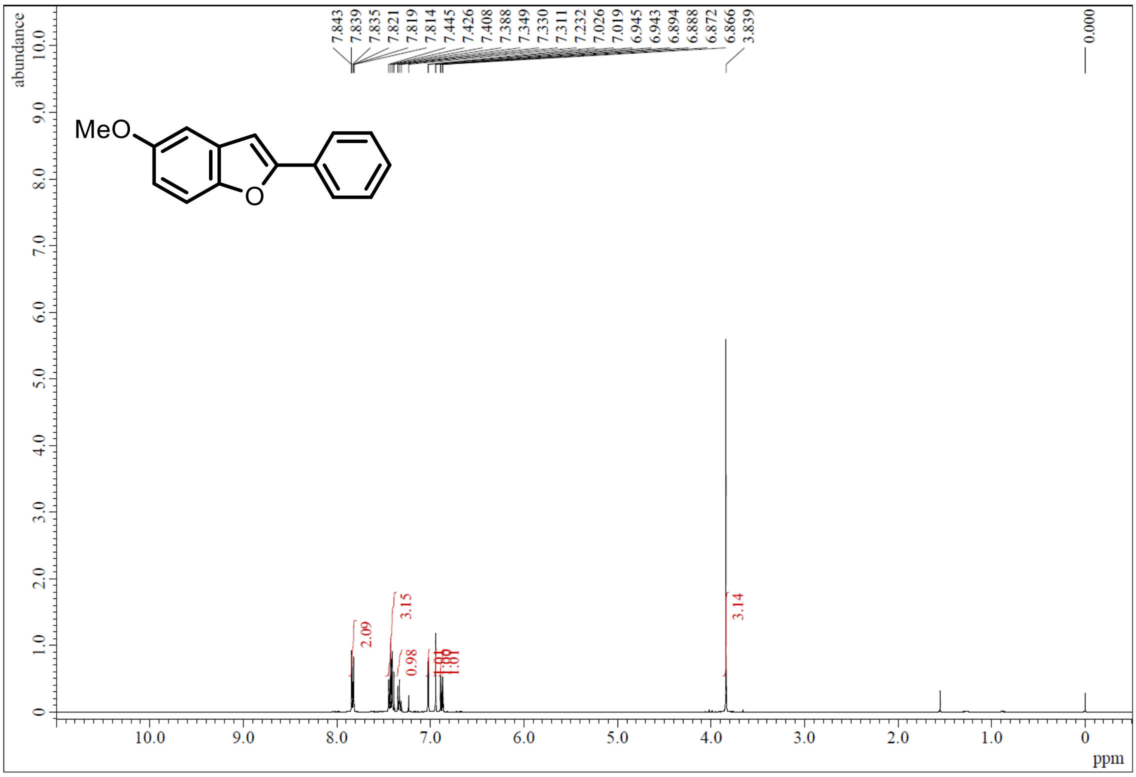

^{13}C-NMR of **13j**

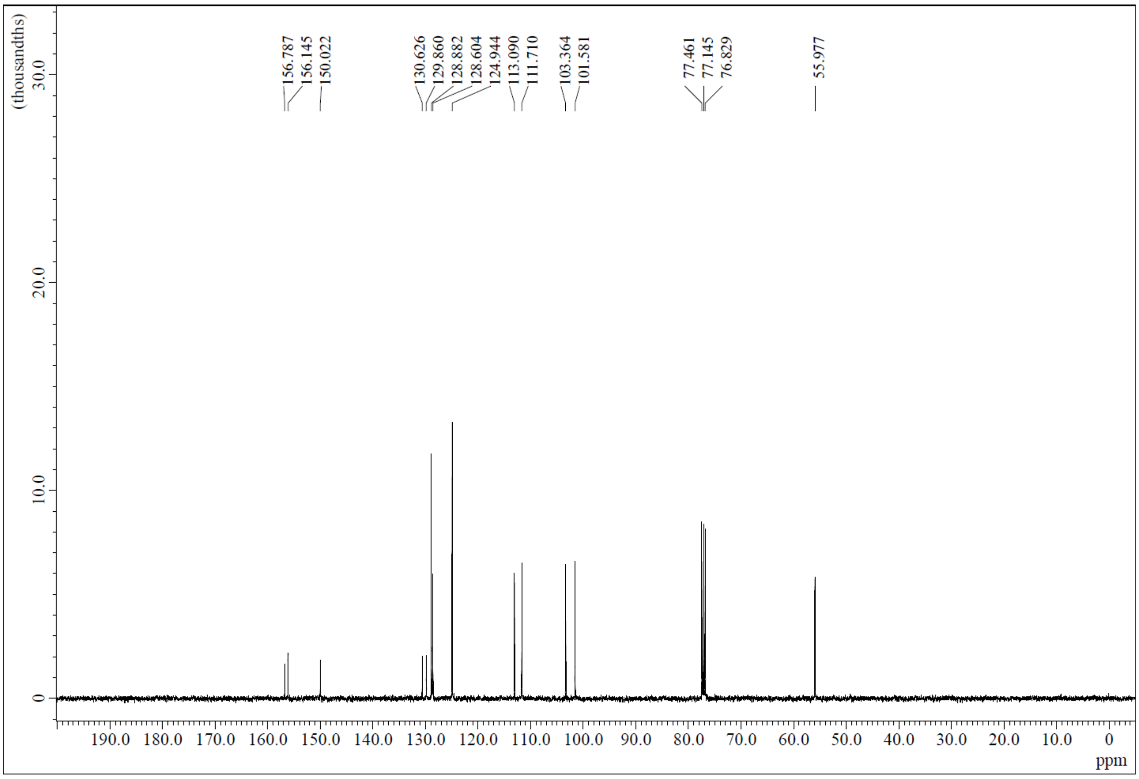

Figure S10. NMR spectra of **13k**.

^{1}H-NMR of **13k**

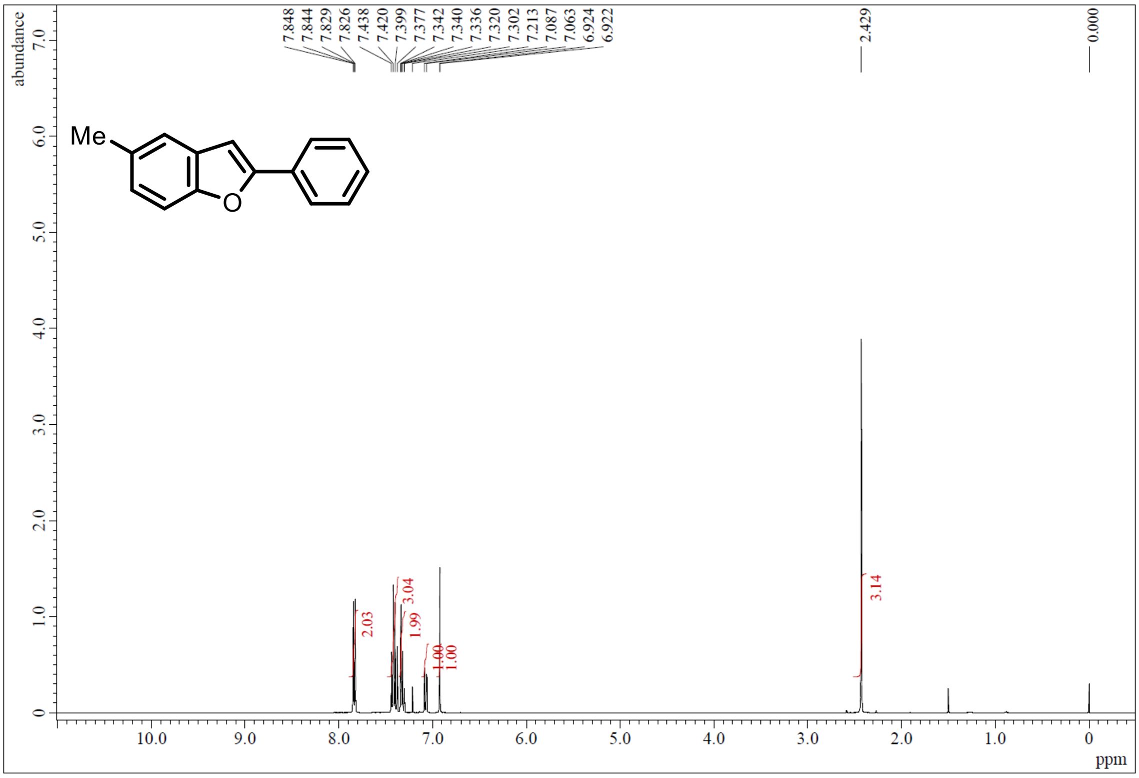

^{13}C-NMR of **13k**

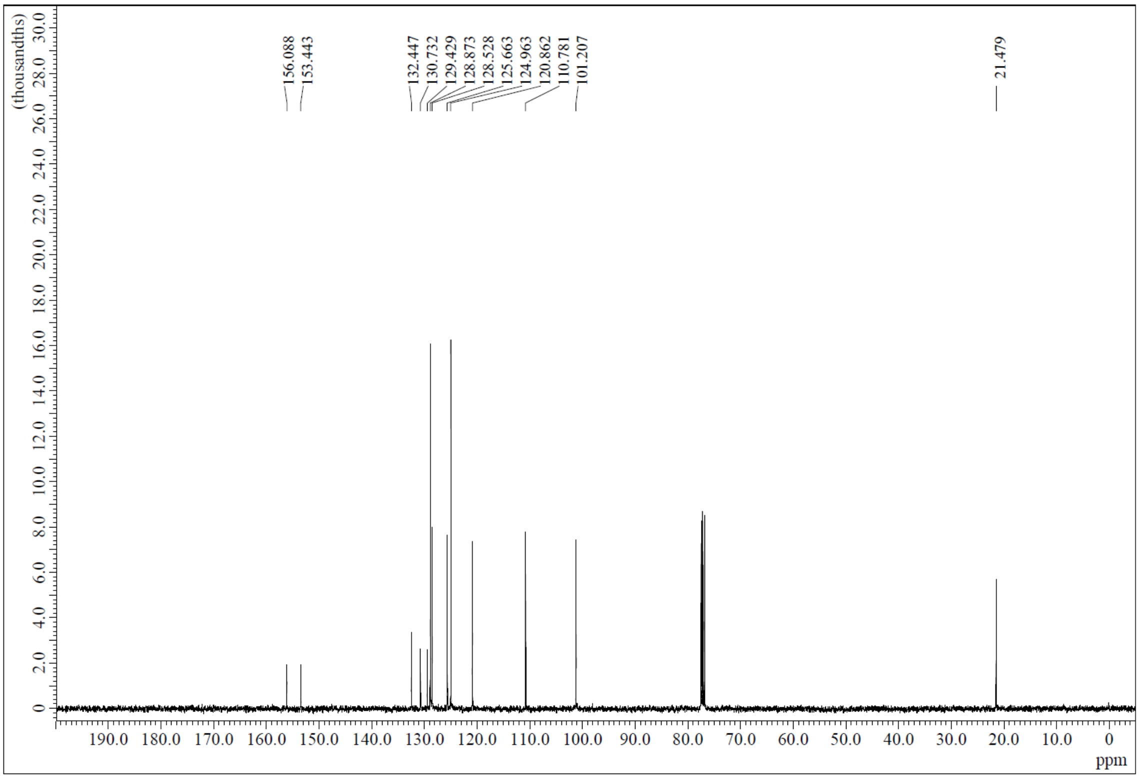

Figure S11. NMR spectra of **13l**.

^{1}H-NMR of **13l**

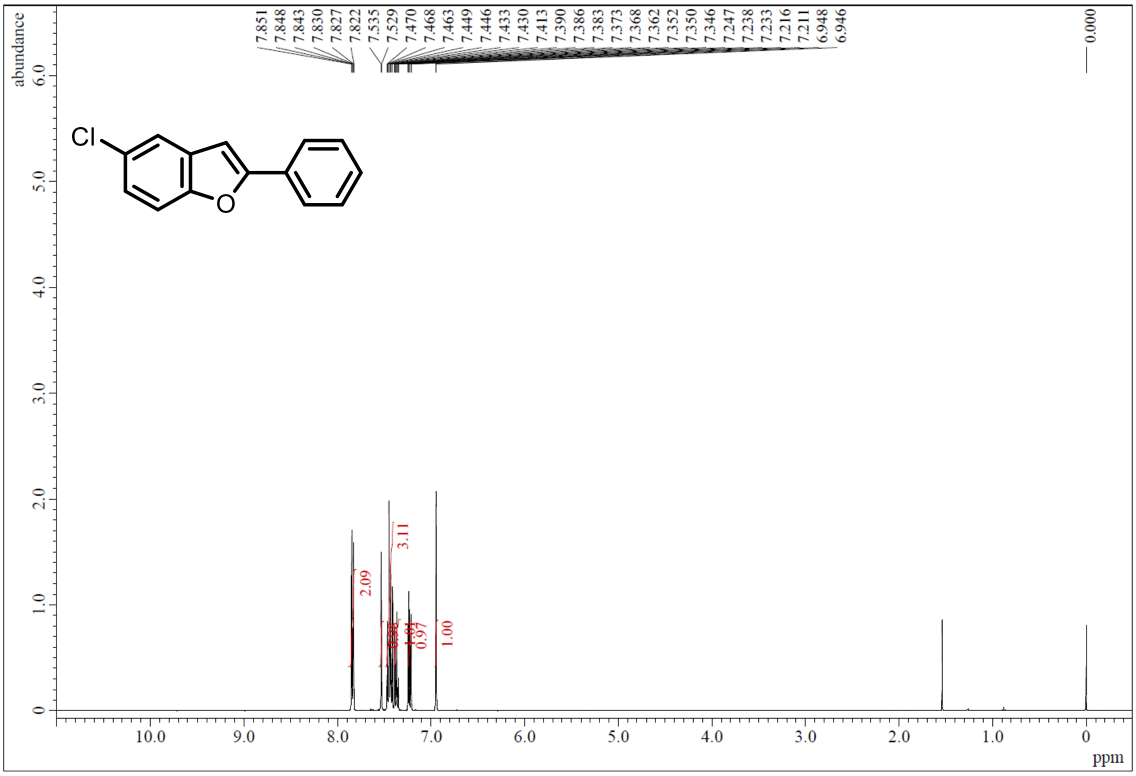

^{13}C-NMR of **13l**

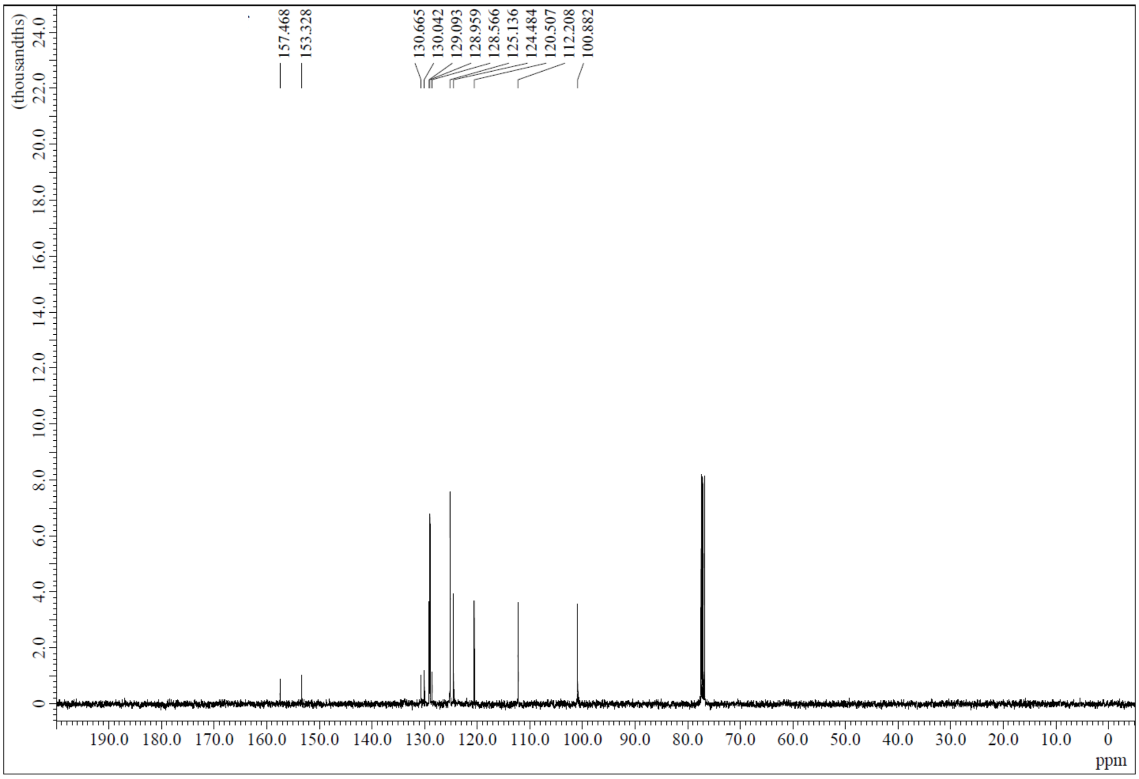

Figure S12. NMR spectra of **13m**.

^{1}H-NMR of **13m**

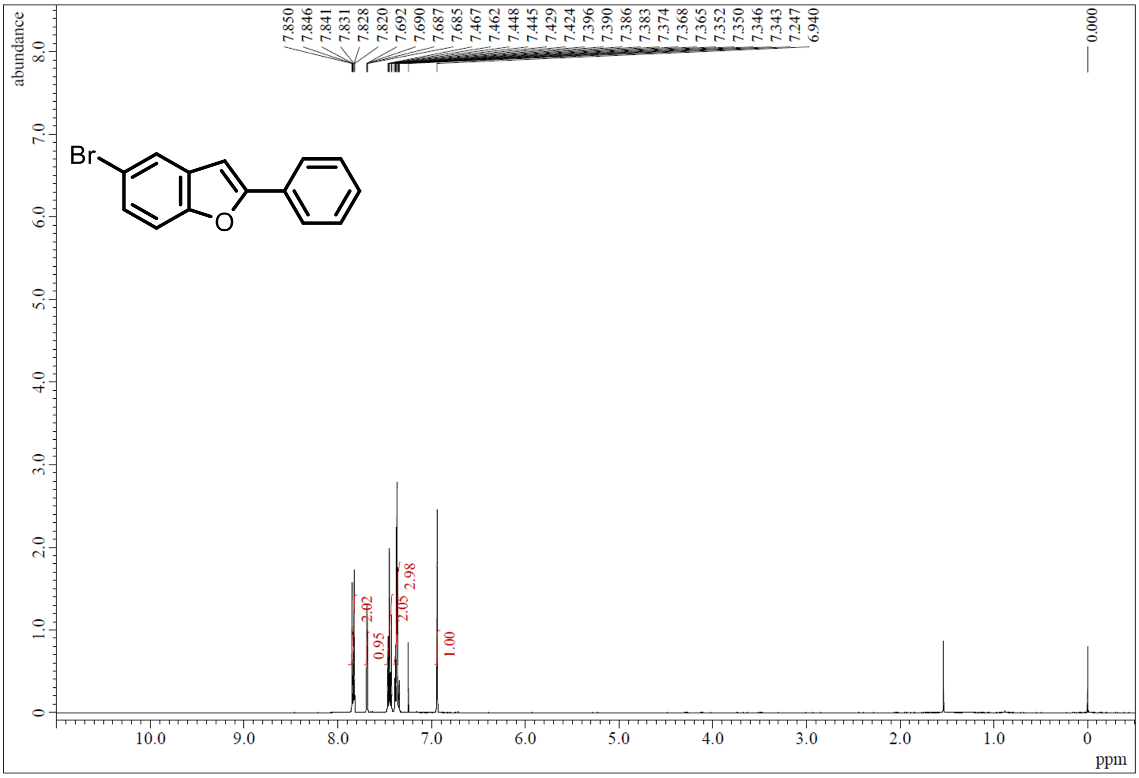

^{13}C-NMR of **13m**

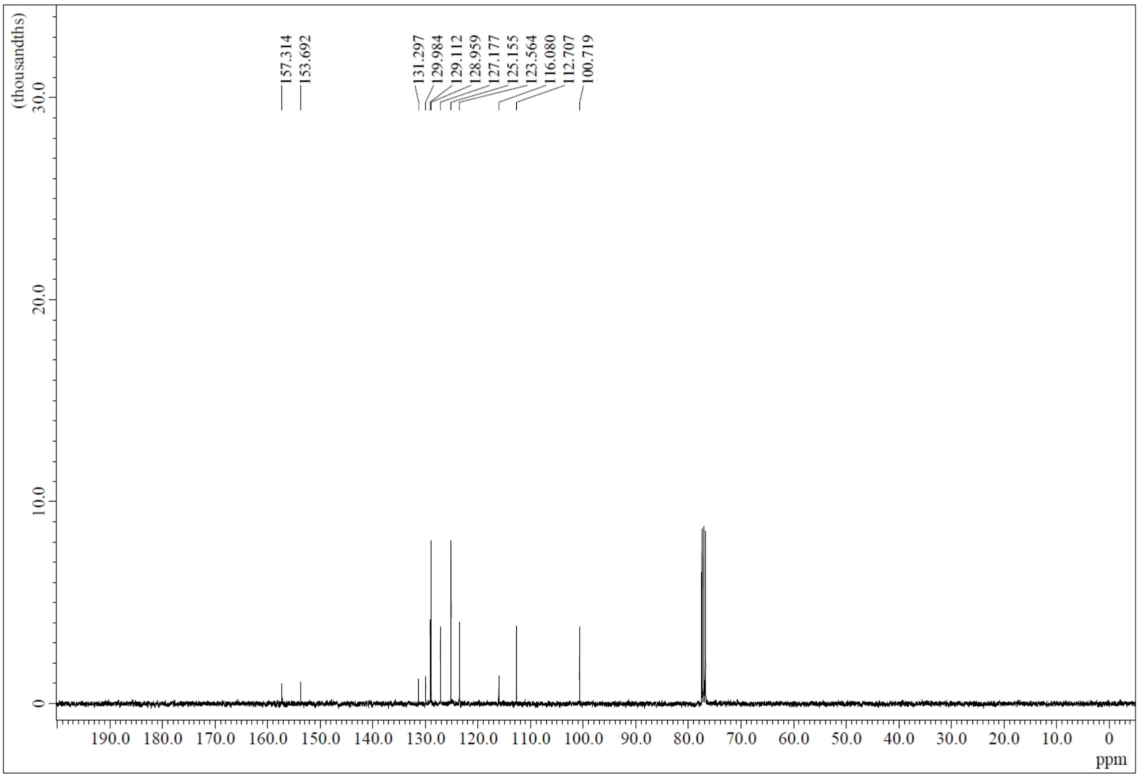

Figure S13. NMR spectra of **13n**.

^{1}H-NMR of **13n**

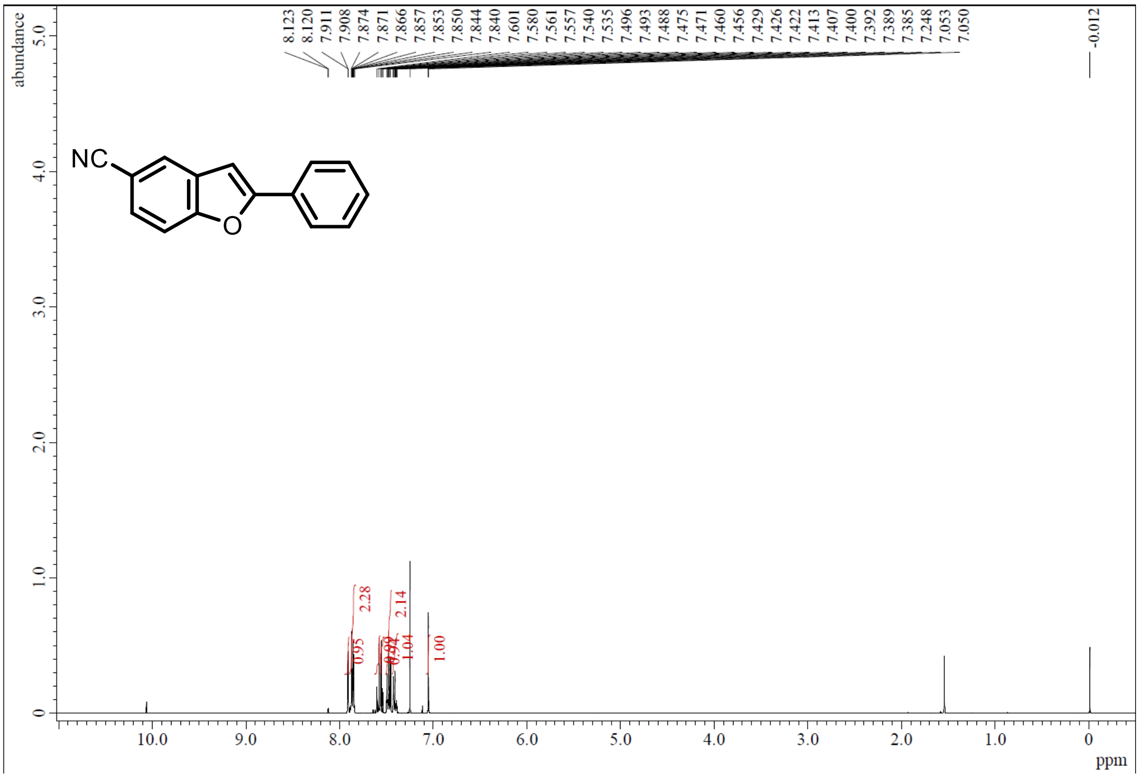

^{13}C-NMR of **13n**

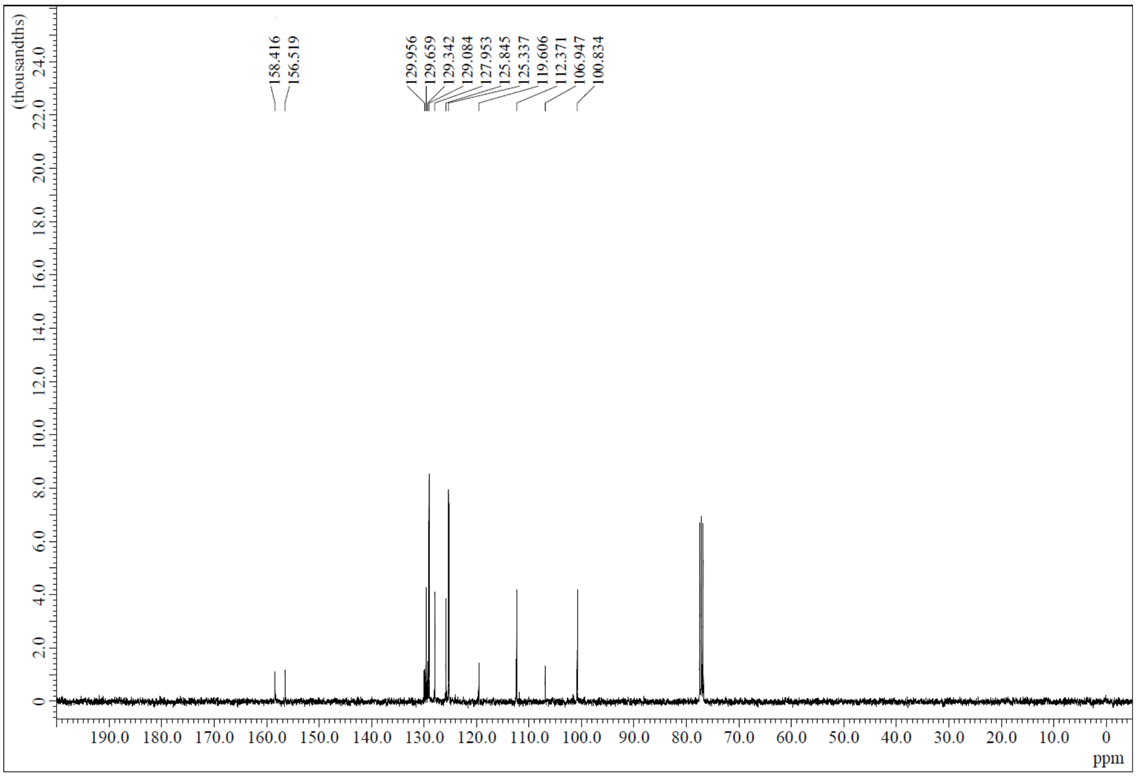

Supplement: Supplementary file 1 [file molecules-26-00097-s001.zip › supporting/Supplementary Information _201128.pdf]
